# Intergenerational and transgenerational effects of endocrine-disrupting chemicals in the offspring brain development and behavior

**DOI:** 10.3389/fendo.2025.1571689

**Published:** 2025-05-21

**Authors:** Glaecir Roseni Mundstock Dias, Fabiana Cardoso Vilela Giusti, Cíntia Onofra de Novais, Maria Aparecida Lima de Oliveira, Alexandre Giusti Paiva, Bruna Kalil-Cutti, Megan M. Mahoney, Jones Bernardes Graceli

**Affiliations:** ^1^ Postgraduate Program in Endocrinology, Faculty of Medicine, Federal University of Rio de Janeiro, Precision Medicine Research Center, Carlos Chagas Filho Institute of Biophysics, Federal University of Rio de Janeiro, Rio de Janeiro, RJ, Brazil; ^2^ Institute of Biomedical Sciences, Federal University of Alfenas, Alfenas, MG, Brazil; ^3^ School of Medicine, Faculty of Science for Health, Federal University of Rondonópolis, Rondonópolis, MT, Brazil; ^4^ Comparative Biosciences, University of Illinois System, Urbana, IL, United States; ^5^ Department of Morphology, Health Sciences Center, Federal University of Espírito Santo, Vitória, ES, Brazil; ^6^ Animal Science, School of Agricultural Sciences, Southern Illinois University, Carbondale, IL, United States

**Keywords:** endocrine-disrupting chemicals, behavior, BPA, bps, BPF, BPAF, phthalates, vinclozolin

## Abstract

Endocrine-disrupting chemicals (EDCs) are a group of substances that can alter normal body functioning by disrupting the various patterns of hormone secretion and action. Some of these substances are used as plasticizers (e.g., bisphenols and phthalates) and agrochemicals (e.g., vinclozolin). EDC exposure can occur by many routes, including oral by contaminated food and water, through the skin, inhalation, and by placental transfer from mother to fetus or mother to infant (via lactation). The increase in EDCs used by the industry has strongly impacted our health. An increasing number of scientific works have reported the effects of EDCs on cancer development, metabolism, heart disease, and fertility. Most recently, studies on EDCs effects on behavior and the developing brain are raising major concerns related to the formation of sex differences and to the increased prevalence of neuropsychiatric disorders. In this review, we highlight the recent findings of the effects of pre-, peri-, and postnatal exposure to the three well-studied EDCs (i.e., bisphenols (BPA, BPS, BPF, and BPAF), phthalates (DBP, BBP, DEHP, and DiPeP), and vinclozolin (VIN)) on developing brain and behavior across generations in experimental animals.

## 1 Introduction

Increased industrial advances correlate with substantial changes in the chemical environment resulting from new industrial and agricultural procedures initiated over the past 40 years (1, 2). Synthetic chemicals used in a variety of industrial and agricultural applications are leading to widespread environmental contamination (2, 3). Although the intended uses of pesticides, solvents, and other chemicals are favorable, the effects on the environment and human health are a global concern (1, 3). A subclass of these xenobiotics is called endocrine-disrupting chemicals (EDCs) (4, 5). EDCs are defined by the World Health Organization (WHO) as “an exogenous substance or mixture that alters function(s) of the endocrine system and consequently causes adverse health effects in an intact organism, or its progeny, or (sub)populations” (3, 6). EDCs are known to display nonmonotonic dose-response curves because they interact with hormones and activate their receptors in a nonlinear fashion, and this leads to a U-shaped or inverted U-shaped curve (3, 7, 8). EDCs could act through genomic and nongenomic nuclear receptor pathways, nonsteroidal receptors, ion channels, and additional mechanisms that could be associated with abnormal growth, inflammation, transgenerational effects, or alterations in epigenetic control, enzymes, and transcriptional coactivators, among other outcomes (7).

EDCs are mainly used as plasticizers (e.g., bisphenols and phthalates), agrochemicals (e.g., vinclozolin), pharmaceutical drugs (e.g., contraceptives), and some can also be naturally produced by plants (e.g., phytoestrogens) (9). EDC exposure might occur by many routes, including oral by contaminated food and water, through the skin (e.g., by insect repellent, cosmetics, and sunscreen), by inhalation, and by placental transfer from mother to fetus or mother to infant (via lactation) (7). Increased industrial and agricultural advances and use of personal care products, among other factors, correlate with substantial changes in the production and use of synthetic chemicals, as well as their improper disposal, and have led to widespread environmental contamination and exposure to different chemicals with EDC activity (1).

The brain plays a critical role in the integration of neuroendocrine function to maintain the body’s homeostasis. It is a frequently reported target of disruption by EDC exposure, with alterations that are commonly influenced by gestational and adult EDC exposure (10, 11). The hypothalamus, hippocampus, prefrontal cortex, and other areas that integrate neuroendocrine activity can be affected by EDC exposure (7). Fetal (*in utero*) and/or newborn exposure to EDCs via placental transport or lactation are of particular concern, as the developing brain is in a state of rapid growth, with neurogenesis, neuronal migration, and differentiation (10, 11). Errors during these processes will have lasting impacts on neural activity, behavioral outcomes, learning and memory, and other factors that can impact health, fitness, and quality of life (11, 12).

The ability of an external EDC to induce an intergenerational and/or transgenerational phenotype requires epigenetic (i.e., DNA methylation, histone modification, and microRNA expression) phenomena mediated through germline modifications (13–16). Thus, abnormal neurogenesis, synaptic connectivity, neurotransmitter or neuropeptide expression, neuronal apoptosis, migration, and irregular differentiation are linked with important changes to physiological systems, including behavior, learning, and memory that could be transmitted inter- and transgenerationally (10, 17), leading to major concerns related to the formation of sex differences and to the increased prevalence of neuropsychiatric disorders (18).

Although there may be hundreds or more environmental chemicals with EDC activity, some classes of these chemicals have been studied in more detail and few EDCs studies have reported intergenerational and transgenerational consequences. In this review, we selected three classes of EDCs based on the knowledge of their direct brain effects associated with motor behavior, anxiety, depression, and cognition, in addition to effects related to sociosexual behavior, namely (1) Bisphenols: 2,2-Bis(4-hydroxyphenyl) propane, also known as 4,4′-Isopropylidenediphenol and Bisphenol A (BPA), dioxydiphenylsulfone or Bisphenol S (BPS), 4,4′-dihydroxydiphenylmethane or Bisphenol F (BPF), and 4,4´-(Hexafluoroisopropylidene)diphenol or Bisphenol AF (BPAF); (2) phthalates: dibutyl phthalate (DBP), benzyl butyl phthalate (BBP), Di-(2-ethylhexyl) phthalate (DEHP), and diisopentyl phthalate (DiPeP); and (3) vinclozolin (VIN). Safety level and main actions in the endocrine system signaling of the BPA, DEHP, and VIN are shown in **Table 1**.

**Table 1:** Main EDC actions in the rodent endocrine system signaling.

EDC	Safety dose	Main actions	References
BPA	50 µg/kg/day (SL/US-EPA) 0.2 ng kg/day (TDI/EFSA) 50 mg/kg/day (NOAEL)	+ ERα and - ERβ ↓ Steroidogenesis ↑ Adipogenesis - β3 integrin/c-Src/MAPK/TR-β1 pathway	(19–28)
Phthalates (DEHP)	0.22 – 0.44 mg/kg/day (SL-TDI) 20 mg/kg/d (NOAEL)	Deregulates PPARs action + ERs ↓ Steroidogenesis + Ras/Akt/TRHr pathway	(29–36)
Vinclozolin	4 mg/kg bw/day (NOAEL) 6 mg/kg/day (NOAEL in acute dietary) 1.2 mg/kg/day (NOAEL in chronic dietary) 0,005 mg/kg bw/day (ADI)	Antiandrogen ↓ GnRH in neurons ↑ Calbindin expression in the ventral POA/AH ↓ Number of GnRH neurons selectively in the region of the OVLT	(4, 37–40)

+: Agonist; -: Antagonist; ↑: Increase; ↓: Decrease; ADI, acceptable daily intake; BPA, Bisphenol A; EDC, Endocrine-disrupting chemical; EFSA, European Food Safety Authority; ERα, Estrogen receptor alpha; ERβ, Estrogen receptor beta; ERs, Estrogen receptors; Src, Steroid receptor coactivator; TR-β1, Thyroid hormone receptor – beta; GnRH, gonadotrophin-releasing hormone; MAPK, Mitogen-activated protein kinases; NOAEL, No-observed-adverse-effect level; OVLT, organum vasculosum of the lamina terminalis; SL, Safety level; TDI, Tolerable daily intake; ventral POA/AH, ventral preoptic/anterior hypothalamic area; Rodent, rat and/or mouse.

**Figure 1:**
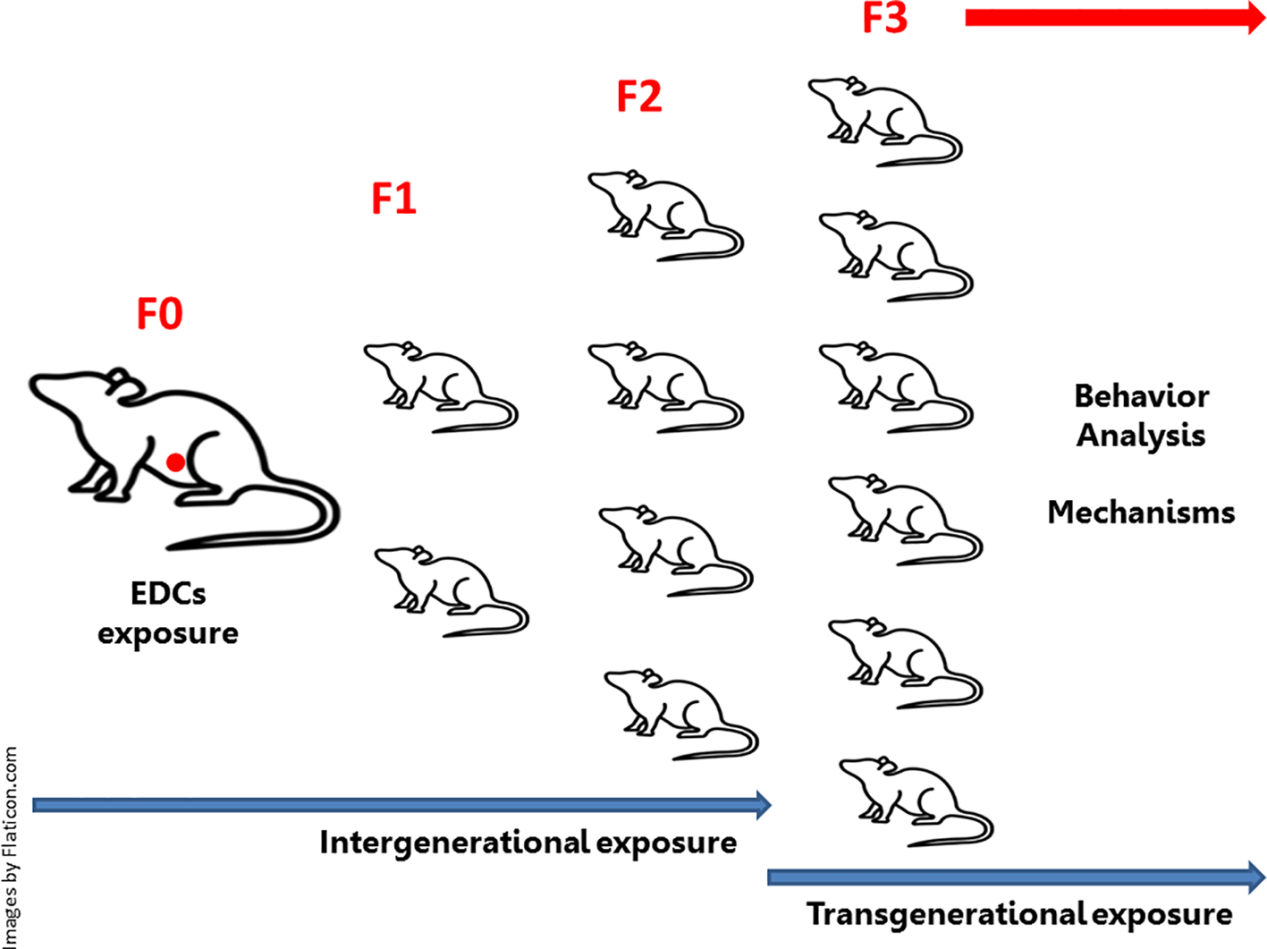
Intergenerational and transgenerational effects of endocrine-disrupting chemicals (EDCs): Embryonic exposure involves exposure of the F0 generation in female pregnancy, the F1 generation embryo (marked in red in the diagram), and the germline of the F2 generation. This intergenerational exposure indicates that the phenotypes of the F0-F2 generations may be due to direct exposure to EDCs. In embryonic exposure, this requires at least one F3 generation to be investigated, as this is the first generation not directly exposed to EDCs (transgenerational exposure). If exposure occurs in non-pregnant (F0) males or females (not represented in the diagram), there will be exposure of the germline of the F1 generation (intergenerational exposure). In this case, the F2 generation will represent the first generation not directly exposed to EDCs (transgenerational exposure).

Articles were selected using the PubMed database. There was no exclusion based on publication year selection of publication time. We utilized the following inclusion criteria: studies testing treatment with EDCs *in vivo* in mammals’ models. We included any dose, duration and life stage of exposure. Only articles that directly described the relationship between dose or exposure and effect were included. Within those criteria, articles studying the three classes of EDCs (1-Bisphenols: BPA, BPS, BPF and BPAF. 2-Phthalates: DBP, BBP, DEHP, DiPeP and 3-VIN) in relation to the direct effects of these classes of EDCs in gestational exposure on the brain functions of mothers and offspring linked to these functions, as well as their intergenerational and transgenerational consequences, were included (**Figure 1**). Each original study cited was critically evaluated for appropriateness of the history of journal quality, indexing, model, the use of adequate controls (negative and positive), the range of dosages tested, methodology, and statistical methods used. No article was excluded based on a positive or negative effect of the EDCs exposure and there was no selection based on target organ or tissue. Articles not written in English were excluded.

## 2 Intergenerational effects of EDCs

### 2.1 Bisphenols exposure

#### 2.1.1 Bisphenol A

BPA is a synthetic chemical used in several arrays of manufacturing, such as food packaging, toys, resins used in the lining of many canned foods and drinks, eyeglass lenses, sports safety equipment, dental monomers, and medical equipment, such as tubing (41). BPA may leach into food, water, or medical fluid tubing under physical manipulation or repetitive use. For children, the estimated exposure ranges from 0.01 to 13 µg/kg/day, with the highest for children who were bottle fed; for adults, the highest estimated exposure was 4.2 µg/kg/day (42, 43). Thermal paper, as used in credit card receipt printers and other types of retail applications, represents an additional source of BPA (20 mg/g paper) as a reactant in the process of heat printing (44, 45). In 2015-2016, the concentrations of BPA in the urine of US women aged 16 to 49 years was 6 µg/L (46). Currently, the United States Environmental Protection Agency (US-EPA) safety level (SL) of BPA is set at 50 µg/kg/day, whereas the European Food Safety Authority’s (EFSA) temporary tolerable daily intake (TDI) was recently lowered to 0.2 ng/kg/day (22, 41, 47) (**Table 1**).

#### 2.1.2 Main mechanism actions of BPA

**Figure 2:**
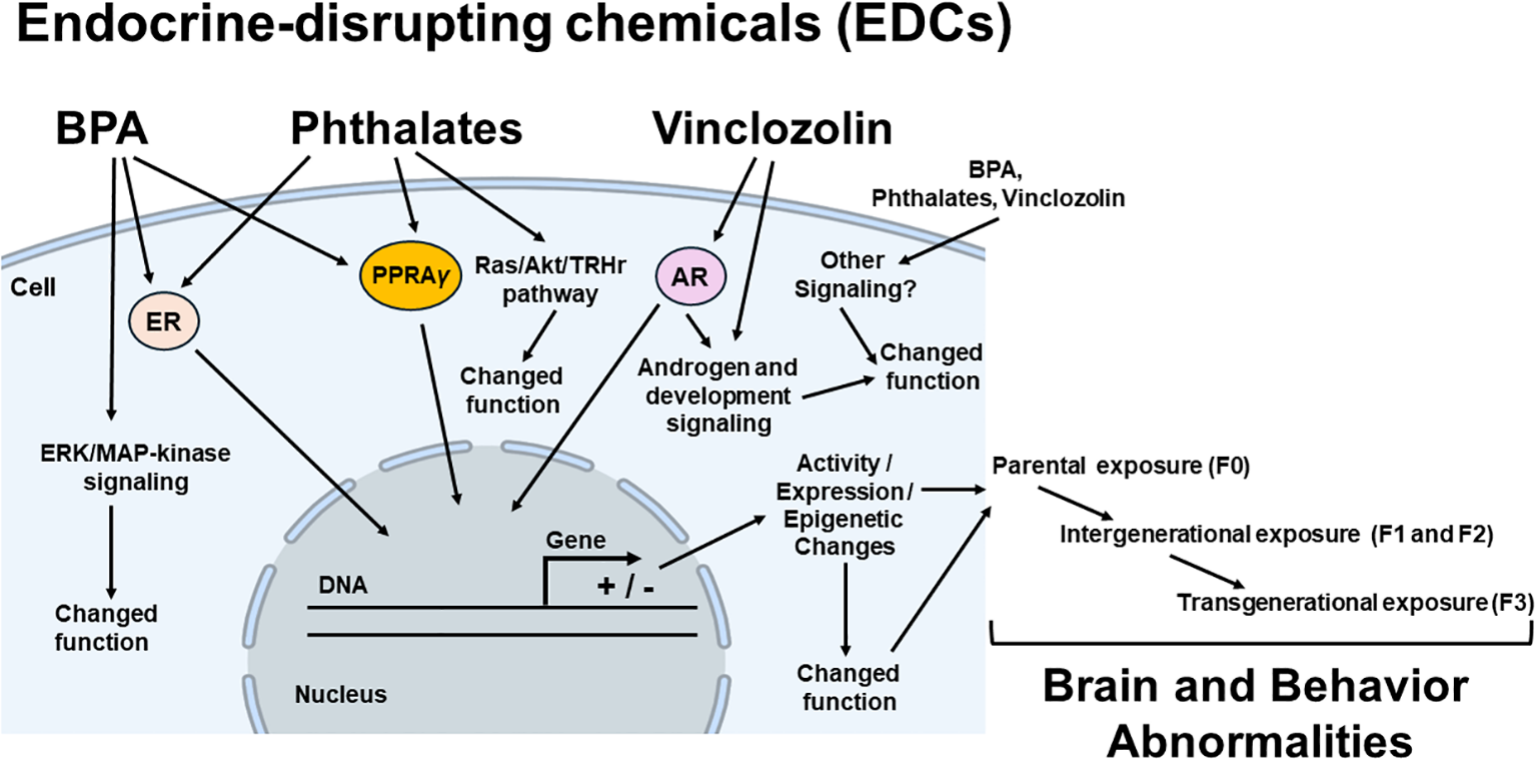
Possible mechanisms of endocrine disrupting chemicals (EDCs) in brain and behavior abnormalities. Potential mediators (intracellular receptors, enzymes and different pathways, epigenetic mechanisms), the EDCs effects of which (brain and behavior abnormalities) have been demonstrated to be induced for each class of EDC, are linked by arrows to the EDC classes in parental (F0), there will be exposure of the germline of the F1 generation (intergenerational exposure). In this case, the F2 generation will represent the first generation not directly exposed to EDCs (transgenerational exposure). BPA, bisphenol A; ER, estrogen receptor; PPARγ, peroxisome proliferator-activated receptor gamma; AR, androgen receptor; ERK/MAPK pathway (extracellular signal-regulated kinase/mitogen-activated protein kinase), Ras, small guanosine triphosphatases (GTPases) from Ras superfamily;/Akt (protein kinase B)/TRHr, thyrotropin-releasing hormone receptor. + stimulate and – inhibit gene expression.

Studies showed that BPA acts like an estrogen agonist, leading to adverse EDC effects (19, 26) (**Figure 2**). Data support that BPA-dependent estrogenic activity occurs through the estrogen receptor 1 (Esr1, formerly known as ERα)-mediated extranuclear signals activation that results in the ERK/MAP-kinase (MAPK) and AKT (or protein kinase B-PKB) phosphorylation (28). However, BPA also acts as an estrogen (E2) antagonist, preventing estrogen receptor 2 (Esr2, formerly known as ERβ) from signaling its nuclear receptor to its downstream signaling targets (i.e., p38/MAPK) (20). It is the consensus that the BPA deregulates enzymatic pathways involved in the steroid hormone biosynthesis and/or metabolism (27) by decreasing the expression of cytochrome P450 side-chain cleavage (Cyp11a1) and steroidogenic acute regulatory protein (StAR) (23). BPA also acts as an obesogenic chemical, changing early adipogenesis by modulating adipocyte hypertrophy and overexpression of lipogenic genes, including peroxisome proliferator-activated receptor gamma (PPARγ) (a nuclear receptor that acts as a master regulator of adipogenesis, which dysregulation is involved in the onset of diabetes and obesity), sterol regulatory element-binding protein 1C, lipoprotein lipase (LPL), and fatty acid synthase, leading to obesity and other metabolic dysfunctions (25). In addition, BPA can alter the β3 integrin/c-Src (Steroid receptor coactivator)/MAPK/TR-β1 (thyroid hormone receptor - beta 1) pathway, suppressing thyroid hormone receptor (THR)-mediated transcription, and may influence thyroid hormone (TH) effects by extranuclear mechanisms (24).

#### 2.1.3 Perinatal bisphenols exposure and the impact on maternal and offspring behaviors

##### 2.1.3.1 Bisphenol A

Prenatal exposure to a low dose of BPA (0.1 ppm in drinking water given to dams during the final week of gestation) dysregulates emotional, learning, and exploratory behaviors in 6-9-week-old offspring. Specifically, it impairs rearing behavior in females on the Open-field Test and increases immobility on the Forced Swimming Test in male rats. These findings indicate that exploratory and depression-like behavior can be affected by only 1 week exposure to relatively low-dose BPA at the late prenatal period in a sex-dependent manner (48). Another study focused on hypothalamus-pituitary-adrenal (HPA) hyperactivity in male rats perinatally exposed to environmental-dose BPA. When female breeders were orally administered 2 µg/kg/day of BPA from gestational day 10 (GD 10) to postnatal day 7 (PND 7), their offspring (PND 80) showed anxiety-like behavior in the Open-field and Light: Dark Test and depressive-like behavior in the Forced Swim Test (FST) (49).

Additionally, there are sex differences in the Elevated Plus Maze (EPM), a test that measures anxiety-like behaviors, and the FST behaviors in adult rats prenatally exposed to BPA. Female rats were exposed to BPA at 5, 50, 500, or 5000 μg/kg/day through gestation and lactation, and the behavior of the offspring was examined. No effect of BPA was observed in the Morris Water Maze, a test of spatial memory. Nonetheless, in the EPM and FST, the low dose (5 μg/kg) of BPA eliminated the sex differences found between controls (50). Interestingly, Xin et al. (51) investigated the molecular basis associated with BPA-induced behavioral changes in first (F1) and second (F2) generations. For this, C57BL/6J dams were treated with BPA from preconception until lactation through the diet at 10 μg/kg/day or 10 mg/kg/day. As adults, F1 male offspring exhibited increased depressive-like behavior, evaluated by the FST, while females were unaffected, which is in contrast to other studies (50). The behavioral changes found in the males were limited to the F1 generation and were not associated with altered maternal care. In addition, these alterations were due to sex-specific disruptions in the serotonergic system and/or sex steroid biogenesis in male offspring.

In a study in mice, BPA was orally administered at a daily dose of 5 μg/kg body weight to F0 pregnant dams from the first day of pregnancy (GD 1) until the last day of lactation (LD 21). The treatment continued in the F1 offspring from weaning (PND 21) to adulthood (PND 100), while F2 offspring were not exposed. BPA exposure decreased maternal behavior in F1 dams and induced changes in sucrose preference in F2 juveniles and in salt and fat solution intakes in F2 adults (52), demonstrating the deterministic effect of altered maternal care induced by BPA on offspring health. Postnatal exposure to low doses of BPA can also affect many emotional conditions in adulthood. For example, female Wistar rat dams were exposed to a low dose of BPA (24 μg/kg/day) during the first 7 days after giving birth, and their male offspring (6–9 weeks old) exhibited increased rearing duration in the Open-field Test. On the other hand, in the EPM, postnatal BPA exposure did not enhance anxiety-like behavior in males; rather, it was associated with an anxiolytic-like effect in females. In the FST, there was an increase in the immobility time associated with a reduction of latency to immobility in BPA rats, indicating that the depression-like responses were clearly enhanced by the postnatal exposure (53).

Regarding the effect of perinatal exposure to BPA on anxiety-like and depression-like behaviors in adulthood, a study tested female and male adult mice (PND 56) following gestational (GD 7 to GD 20) or lactational (PND 1 to PND 14) exposure to BPA (0.4 or 4 mg/kg/d, orally administered). Females with gestational exposure exhibited an increase in anxiety-like behavior in the Open-field, Light: Dark transition task, mirror maze, and EPM tasks. Furthermore, females with lactational exposure and the males with gestational exposure exhibited anxiogenic-like behavior. However, males exposed to BPA during lactation exhibited anxiogenic-like behavior only in the EPM. The results of the FST showed that gestational exposure markedly increased the immobility time in both sexes, and the same effect was induced by lactational exposure only with 4 mg/kg/d BPA (54). In another study, BPA was administered orally to a group of female rats (40 µg/kg) from 10 days before mating until the weaning of the pups. In a second group, BPA was given at a higher dosage (400 µg/kg) during a critical period for brain organization, i.e., from GD 14 to PND 6. The offspring of the treated mothers were tested in the Hole Board and the EPM at PND 85. In general, the factor analysis indicated that in the treated group, the motivation to explore anxiety-like behaviors is reduced in males, while in females, only motor activity and motivation to explore are reduced, indicating a clear sex-dependent effect of BPA on anxiety and depressive-like behavior (55).

Interestingly, perinatal BPA exposure from GD 10 until PND 20 in rats (4, 40, or 400 mg/kg/day orally) revealed that BPA lengthened immobility time in the dark phase in female offspring during spontaneous activity. In 4-week-old males that received BPA at high doses (40 and 400 mg/kg/day), there was a significantly higher number of avoidance responses in the Shuttle box Avoidance Test. This test is used to measure the association of an adverse experience with a particular stimulus. However, at 8 weeks of age, males treated with the lower dose (4 mg/kg/day) showed significantly lower responses, when compared to the control group, in the Avoidance Test, showing that a relatively small dose of BPA can affect neurobehavioral development. In addition, in the Open-field Test, only the lower dose (4 mg/kg/day) increased the percentage of grooming in the BPA-exposed male rat compared to the control rat (56).

In a cross-fostered study, the outcome of pre- and postnatal exposure to BPA in female offspring was similar. Pregnant mice were orally treated with BPA (10 μg/kg/day) starting from the last week of pregnancy to the first postpartum week. At birth, the litters were cross-fostered to different dams to differentiate among the effects of pre- and postnatal exposure. Pre- and postnatally exposed offspring underwent testing for anxiety-related behaviors as juveniles (the novelty test) and as adults (Open-field and EPM Tests). At both testing ages, pre- and postnatally exposed females had increased anxiety-like behaviors and were less likely to explore a novel environment relative to the control females (57). The effects of developmental BPA exposure can decline from adolescence to adulthood. For instance, rats were given exposure to BPA via drinking water (1 mg/L) from GD 6 to PND 40 and then tested for anxiety-like behaviors. Animals were tested after weaning but prior to puberty (PND24-28) using the Light: Dark box and EPM. Adult behavior was assessed from PND 60–70 using the EPM. The results revealed that early life exposure to BPA elevates juvenile anxiety-like behavior in rats of both sexes and causes loss of sexual dimorphism in adult exploratory behavior. The authors suggested that behavioral impacts of BPA can manifest during adolescence but wane in adulthood (58).

Moreover, long-term neurobehavioral effects of developmental BPA exposure may be reversible in the adolescent period. For instance, BPA administered orally in female rats (40 mg/kg/day) during the entire period of pregnancy and lactation increased anxiety-like behaviors in the Open-field Test in female offspring at PNDs 38-45. These effects were reversed by basolateral amygdala (BLA) treatment with 5-aza-deoxycytidine (a DNA methylation inhibitor), and this drug restored the increased synaptic transmission in the BLA via improving the GABAergic system. Thus, the authors suggested that the overexpression of DNA methyltransferase 1 (DNMT1) in the BLA is responsible for the etiology of anxiety associated with BPA exposure via GABAergic disinhibition (59).

In mice, prenatal exposure (GD 0-19) to BPA (2, 20, and 200 μg/kg/day) induced sex-specific effects on social and anxiety-like behavior, leading to disruption of sexually dimorphic behaviors at PND 30-70. Although postnatal maternal care was altered in mothers treated with BPA during pregnancy, the effects of BPA *in utero* were found to not be mediated by maternal care. However, these data suggest that increased maternal care may partially attenuate the effects of *in utero* BPA on DNA methylation. Overall, low-dose prenatal BPA exposure induces lasting epigenetic disruption in the brain that possibly underlies enduring effects of BPA on brain function and behavior. BPA altered mRNA levels of epigenetic regulators DNA methyltransferase (DNMT) 1 and DNMT3A in the juvenile cortex and hypothalamus. Importantly, changes in Esr1 and DNMT expression in the cortex (males) and hypothalamus (females) were associated with DNA methylation changes in the Esr1 gene (60).

To clarify mechanisms involved in the anxiety-like behavior of offspring prenatally exposed to BPA, pregnant Wistar rats were exposed to BPA in drinking water (25 µg/L, 250 µg/L, or 2.5 mg/L) from GD 9 to GD 24. Prenatal BPA exposure increased anxiety-like behavior in males and decreased exploratory behavior in both male and female offspring (PND 21-29). These behavioral responses were associated with a downregulation of both BDNF (Brain-Derived Neurotrophic Factor) and CYP19A1 (Aromatase enzyme) genes in males, whereas the expression of both genes was upregulated in the female offspring. Both the male and the female BPA-exposed offspring exhibited elevated levels of DNMT1 protein. The sex-specific alteration in the expression of CYP19A1 and DNMT1 genes suggests that both hormonal and epigenetic dysregulation could underlie the long-term BPA-induced effect on anxiety-like behavior in the offspring (61).

In addition, when rats were orally administered BPA (40 mg/kg/d) during pregnancy and lactation, their female offspring at PND 40–50 developed an anxiety-like phenotype, characterized by hyperactivity of the hypothalamic-pituitary-adrenal (HPA) axis, impaired glucocorticoid receptor (GR)-mediated negative feedback regulation of the HPA axis, altered hippocampal synaptic plasticity, and increased anxiety-like behaviors associated with a reduction in the expression of metabotropic glutamate 2/3 receptors (mGlu2/3 receptors) in the hippocampus (27). Moreover, anxiety-like behavior due to perinatal exposure to BPA may be associated with a decrease in excitatory to inhibitory synaptic density in postnatal male mice. Specifically, following perinatal exposure to BPA (50 µg/kg/d from GD 7 to PND 21), 8-week-old mice tested in the Open-field Test had reduced time spent, number of entries, and distance traveled in the central zone. In the EPM, there was a decrease in time spent and number of entries to the open arms. There was also significant downregulation of synaptophysin but upregulation of gephyrin, a scaffolding protein that clusters and stabilizes receptors at inhibitory synapses, such as glycine and gamma-aminobutyric acid type A (GABA A) receptors, thus leading to a reduction in the excitatory-to-inhibitory protein ratio and synaptic density in postnatal 3- and 8-week-old mice (62).

Further, maternal exposure to BPA (10 or 50 μg/kg/day) during gestation and lactation induces life-long changes that compromise behavior in EPM in both female and male adult rats (63). Likely, maternal BPA diet (50 mg/kg, 2 weeks before mating and during pregnancy and lactation) induces excessive anxiety-like behavior in female juvenile mice (as evaluated in the Open-field and EPM Tests) associated with elevated TNF-α and IL-6 levels, as well as activated microglia and astrocytes in the prefrontal cortex, showing that the perinatal BPA effects may occur at least in part due to neuroimmune activation (64).

Regarding cognitive behavior, it has been shown that daily administration of 4, 40, or 400 μg/kg BPA to the dams throughout pregnancy plus direct oral administration of BPA to the pups from PND1–9 did not affect the performance on the Radial Arm Maze in male or female offspring (65). On the other hand, the effects of prenatal BPA on behavioral responses to a predator odor have been shown to have an amplifying effect on avoidance responses (66). Regarding maternal behavior, BPA exposure (40 or 400 µg/kg/day in cookie) from GD 2 until parturition and later offered to the offspring (PND 1-9) changed performance on a social recognition task in both doses in the pups (PND 26-40) (65). However, in adulthood (PND 90), BPA did not impact behavioral parameters on the EPM test. The dams’ behavior was recorded every 3 min for 90 min (30 observations/night), and 400 µg BPA/kg/day resulted in less licking of pups when compared to females dosed with 40 µg BPA/kg/day. It is worth noting that during the maternal observations, BPA was being administered directly to the pups but not to the dams. Thus, the decrease in licking by the dams may be due to BPA-induced changes in the pups, perhaps by altering behavioral or olfactory cues (67).

Transplacental and lactational exposures of BPA (0.1 mg/kg/day, orally, from GD 3 to PND 20) demonstrated that in the Active Avoidance Test, at 15 weeks of age, BPA-treated offspring showed fewer avoidance responses compared to non-treated rats. Furthermore, BPA-treated offspring increased the number of failures to avoid electrical unconditioned stimuli within 5-sec electrical shock presentation compared with the control offspring. These results indicate that perinatal low-dose BPA exposure irreversibly influences the reception of fear-provoking stimuli (68).

Play behavior of female rats was used to explore the effects of perinatal exposure to a low, environmentally relevant dose of BPA. In this study, dams were exposed to 40 µg/kg/day BPA during pregnancy and lactation. The early administration of BPA was responsible for a significant increase in social exploration investigation as well as a decrease in play with males and social grooming. The authors suggested that the general decrease in playful interactions induced by BPA did not induce a clear masculinization of female behavior but rather defeminized some aspects of female behavior (69). In contrast, a study treated female Sprague-Dawley rats orally with 40 µg/kg/day of BPA from conception to weaning (PND 21) and 400 µg/kg/day of BPA from GD 14 to PND 6. After exposure, social behavior was examined in juvenile male and female offspring. Masculinization of female behavior was observed in two behavioral categories (play with females and sociosexual exploration), an effect probably mediated by the estrogenic activity of BPA in the central nervous system (70).

Furthermore, a range of conflicting results is seen in the literature. A study by Ryan et al. (71) reported no effect on any sexual behavior parameters, Sucrose Preference Test or locomotor activity in response to maternal exposure to low doses of BPA. Similarly, two other studies showed no effect of low doses of BPA exposure in the perinatal period on the social choice (juveniles) and Open-field (adults) Tests (72) or on sexual behavior in pubertal female rats (73). However, another study, in which BPA (40 µg/kg/day) was administered to female rats during pregnancy and lactation, evaluated the agonistic and sexual behavior of adult female and male rats. In BPA-exposed males, the Intruder Test revealed an increase in defensive behavior compared to controls. Male sexual orientation toward a stimulus female was not affected by BPA, whereas the sexual activity test showed a slight impairment of sexual performance due to BPA in terms of latency and frequency of intromissions. In females, BPA produced a small increase in sexual motivation and receptive behavior (74). In addition, males receiving a relatively low dose of BPA (50 μg/kg bw/day orally) perinatally (GD7-PND14) showed persistent deficits in sexual behavior in adulthood. Surprisingly, males that received the highest dose (5 mg/kg/day) were indistinguishable from controls with respect to consummatory sexual behaviors. Nevertheless, these BPA-exposed males had decreased latencies to engage in those behaviors when sexually naïve. Adult female sexual behavior was not affected by early BPA administration at any dose tested (75).

On the other hand, when mouse dams were orally exposed to BPA at doses equivalent to the no observed adverse effect level (5 mg/kg body weight per day) and tolerable daily intake (TDI, 0.05 mg/kg body weight per day) from GD 15 until weaning, female offspring showed an increase in lordosis quotient at the TDI dose only. BPA exposure had no effect on olfactory preference, ability to express male behaviors, or the number of calbindin-positive cells, a sexually dimorphic population of the preoptic area in the hypothalamus. BPA at both doses selectively increased kisspeptin cell number in the preoptic periventricular nucleus of the rostral periventricular area of the third ventricle in adult females. This kisspeptin population is known to play a key role in triggering preovulatory gonadotrophin surges. However, BPA exposure did not affect the number of GnRH-positive cells (gonadotrophin-releasing hormone) or the percentage of kisspeptin appositions to GnRH neurons in the preoptic area.Kisspeptin cell number changes were associated with higher levels of estradiol (E2) at the TDI dose, while levels of LH, estrus cyclicity, ovarian and uterine weights, and fertility remained unaffected. A delay in the time of vaginal opening was observed during the postnatal period at the TDI dose, without any alteration in body growth (76).

Neurobehavioral development was investigated in rats exposed perinatally to BPA from GD 7-PND 7 at doses of 0, 25 µg, 250 µg, 5 mg, or 50 mg/kg bw/day by a behavioral testing battery, including tests for motor activity, sucrose preference, anxiety, and spatial learning. No behavioral effects were seen in male offspring. In the female offspring, exposure to 25 µg/kg bw/day of BPA caused altered spatial learning in the Morris Water Maze (72). Additionally, embryonic exposure to 0, 0.05, 0.5, 5, and 50 mg/kg/day of BPA by oral gavage from GD5 to GD19 impacted the spatial learning and memory of rats in later life. Morris Water Maze performance in male and female offspring at PND 56 showed that exposure to 0.5 mg/kg/day of BPA disrupted the spatial learning ability of rats. However, spatial memory ability could only be affected by exposure to BPA at doses up to 5 mg/kg/day (77). Using these same doses, from GD 9 to GD 20, the effects of maternal BPA exposure on memory and synaptic structure in the hippocampus of male offspring at PND 21 were analyzed. Locomotor activity and emotional behavior in Open-field Test were affected; specifically, increased reference and working memory errors were found in the radial arm maze during the postnatal developing stage. These findings may be involved in synaptic plasticity alteration since maternal BPA exposure had an adverse effect on synaptic structure (78).

Altered profiles of spontaneous novelty seeking, impulsive behavior, and response to amphetamine in rats perinatally exposed to BPA were observed. BPA was administered orally to mother rats from GD0 to pups’ weaning at a concentration of 0.04 mg/kg. The offspring of both sexes were tested during adolescence (PND 35-45) for novelty preference. BPA-exposed females spent significantly less time than did controls in exploration of the novel side, whereas no effect was found in the male group. During adulthood, the same animals were tested for profiles of impulsive behavior in operant chambers provided with two nose-poking holes (delivering either five or one food pellet). A reduced level of impulsive behavior was seen in BPA-treated rats. Animals were then tested for the response to an amphetamine challenge. The drug-induced increase in locomotor activity was significantly less marked in BPA-treated male rats compared to the controls. These findings provide indirect evidence of long-term alterations in monoaminergic brain function after perinatal BPA exposure (79).

Perinatal exposure to low-dose BPA (GD 10 to PND 14, 50 or 500 µg/kg/day, orally) impairs spatial learning and memory in male offspring rats in the Appetite-Motivated Maze Test. In the Maze Test, male offspring of dams exposed to BPA 50, but not those of dams exposed to BPA 500, needed more time to reach the reward (80). Moreover, another study also demonstrated that perinatal exposure to low doses of BPA (40 μg/kg/day) in rats disrupts learning/memory during adulthood in association with DNA methylation of Esr1 in the hippocampus (81). Current findings suggest developmental exposure of rats to BPA (from GD 6 to PND 21 with 2.5, 25, or 2500 μg/kg/day; per orally) may disrupt aspects of spatial navigational learning and memory, which are tested in the Barnes Maze. In adulthood, the 2500 BPA group sniffed more incorrect holes on day 7. The 2500 BPA females were less likely to locate the escape box in the allotted time, whereas 2.5 BPA males showed improved latency compared to control males (82).

In order to investigate the mechanism of action of perinatal exposure to BPA on learning and memory, Xu et al. (83) showed that inhibition in the expression of NMDAR subunits and Esr2 in the hippocampus during the postnatal development stage may be involved in the reduced performance in the Morris Water Maze and passive avoidance test at both PND 21 and PND 56. Additionally, another work investigated the effects of BPA on fear memory and serotonin (5-HT) metabolites in the brain using contextual fear conditioning (FC) and high-performance liquid chromatography (HPLC), respectively, in adults and juvenile mice of both sexes. In female juvenile mice, BPA enhanced fear memory and increased serotonin metabolite (5-HIAA) and 5-HIAA/5-HT levels in the hippocampus, the striatum, the midbrain, the pons, and the medulla oblongata. In contrast, alterations in those areas were much smaller in adult females and in both juvenile and adult males (84). Thus, prenatal and postnatal BPA exposure leads to sex- and dose-dependent changes in emotional and cognitive behaviors, with important effects on anxiety, depression-like behaviors, and learning in offspring. These effects are affected by exposure timing, with critical windows during pregnancy and early postnatal life being particularly sensitive. Furthermore, these behavioral alterations are often linked to epigenetic changes, suggesting long-term impacts on brain function that may persist into adulthood.

##### 2.1.3.2 Bisphenol S

BPS is an analog of BPA that differentiates from BPA by possessing a sulfone group (SO_2_). BPS is also used in the manufacturing of hard plastic and plastic fibers, as well as in curing fast-drying epoxy resin adhesives (85). A study investigated the effects of BPS (2 or 200 µg BPS/kg/day given on a small wafer) on maternal behavior and brain in CD-1 mice exposed during pregnancy and lactation (F0 generation) and in female offspring exposed during gestation and perinatal development (F1 generation). In this study, different effects were observed on F0 and F1 dams for several components of maternal behavior, including time on the nest, time spent on nest-building, latency to retrieve pups, and latency to retrieve the entire litter. F0 dams exposed to the higher dose of BPS spent significantly more time on the nest on PND 14. These results suggest that BPS exposure in the F0 dams induces an extension of a behavior that is generally diminished by this stage of the postpartum period; this may indicate a lack of adjustment in the dam to the changing needs of her pups. In contrast, dams of the F1 generation developmentally exposed to either 2 or 200 µg BPS/kg/day spent more time away from the nest on PND 2 and PND 7. During these early postpartum periods, the pups require protection and constant care for their growth and survival, including attention to thermoregulation and feeding. Thus, the F1 BPS-exposed dams display quantitatively diminished care (86).

Wistar rats born from dams that were BPS-exposed to BPS10 (10 µg/kg/day) or BPS50 (50 µg/kg/day) via intragastric gavage during pregnancy and lactation demonstrated that in adulthood (PND 160), only BPS10 and 50 males had higher anxiety-like behaviors because they spent less time and entered less in the open arms of the EPM. Locomotor activity was not affected (58). Low doses of BPS can alter lactational behaviors and the maternal mammary gland by significantly reducing the fraction of the mammary gland comprised of lobules, the milk-producing units, on PND 21, but not PND 2. BPS also altered the expression of the ERα gene (Esr1) and protein in the mammary gland at PND 21, consistent with early involution. Observations of nursing behavior collected during the lactational period revealed that BPS-treated dams spent significantly more time nursing later in the lactational period, and BPS-treated pups were less likely to initiate nursing. Pup growth and development were also stunted (87).

##### 2.1.3.3 Bisphenol F

BPF is also structurally related to BPA and has been used as a substitute for BPA. However, Ohtani et al. (88) observed behavioral adverse effects in the mice offspring exposed to BPA or BPF in the fetal period. Female C57BL/6 mice were given oral BPA or BPF (0 or 10 mg/kg body weight) daily from gestational day 11.5 to 18.5. The Open-field, EPM, and FST were performed at postnatal week 10. Significant effects of BPF were found in all behavioral tests, especially in females. In the BPF group, anxiety-like behaviors increased when compared to controls. It is known that male mice typically display more anxiety than females. However, after BPF exposure, there was an abolishment of typical sex differences. In addition, BPF increased depressive-like states compared to the controls, which was not the case with BPA; these findings suggest that BPF may induce more negative effects in the fetus than BPA.

##### 2.1.3.4 Bisphenol AF

BPAF is a new chemical structurally related to BPA with stronger estrogenic activity compared to BPA. One study aimed to investigate the potential effects of maternal BPAF exposure during pregnancy on the emotional behaviors of adolescent mice offspring of both sexes. In males, maternal exposure to BPAF (0.4 or 4.0 mg/kg, intragastrically administered daily from GD 1 to GD 19) induced significant anxiety- and depressive-like behaviors, elicited by Open-field Test, Novelty-Suppressed Feeding (NSF) Test, Sucrose Preference Test, Tail Suspension Test and FST. In females, BPAF exposure at 0.4 mg/kg reduced the latency to feeding in the NSF test, while it increased floating time in the FST. Maternal BPAF exposure decreased the recognition index in long-term memory in the novel object recognition test in both sexes, while it only decreased the freezing time of male offspring in the contextual fear conditioning task. Therefore, maternal exposure to BPAF significantly affects emotion-related behaviors in adolescent mice, with the male offspring exhibiting a higher probability of developing symptoms of anxiety and depression and suffering memory impairment compared to females (89).

Intergenerational behavioral data of the bisphenols (BPA, BPS, BPF, and BPAF) are shown in **Supplementary Table S1 (Supplementary material)**.

### 2.2 Phthalates exposure

Phthalates and phthalate esters, like dibutyl phthalate (DBP), diethylhexyl phthalate (DEHP), and diisopentyl phthalate (DiPeP) are a large class of manmade and multifunctional chemicals used as liquid plasticizers found in a wide range of products, including plastics, coatings, toys, cosmetics, and medical tubing (90). DEHP exposure through children’s toys (by sucking or chewing) or other sources was estimated to be up to 85g/kg/day (91, 92). In 2017-2018, the concentrations of DEHP metabolites in the urine of women aged 16 to 49 years was 9 µg/L (46). US-EPA suggests the safety level (SL) of DEHP is set at 0.22 mg/kg/day, whereas the Health Canada-European Medicines Agency suggests the TDI as 0.44 mg/kg/day (**Table 1**) (29, 93).

Intergenerational behavioral data of the phthalates are shown in **Supplementary Table S2 (Supplementary material)**.

#### 2.2.1 Main mechanism actions of Phthalates

It has been described that an important mechanism of action of phthalates is involved with the peroxisome proliferator-activated receptor (PPAR) signaling to deregulate the expression of downstream PPAR-related genes as an obesogenic action (31, 32) (**Figure 2**). Another mechanism of phthalates’ action is the target of ER signaling (35), making phthalates have an “estrogenic” like-activity. However, this activity is weak, and all phthalates’ esters tested are non-estrogenic in female rats, so there are controversies about the action of phthalates through ERs (34, 94). Similar to other EDC activities, phthalates and their metabolites have been suggested to interfere with proper steroidogenesis function, suppressing the expression of steroidogenic enzymes in testis and ovarian tissue (33). Phthalates like DEHP also have thyroid-disrupting effects, activating the Ras/Akt/TRHr pathway and inducing the expression of hepatic enzymes such as Ugt1a1, CYP2b1, Sult1e1, and Sult2b1, leading to metabolic abnormalities (36) (**Figure 2**).

#### 2.2.2 Perinatal phthalates exposure and the impact on maternal and offspring behaviors

##### 2.2.2.1 DEHP

The effects of perinatal (GD 0-PND 10) and adolescent (PND 27-55) exposure to phthalate (DEHP) at dosages of 0, 200, or 1000 μg/kg/day on anxious-like behaviors were evaluated in mice when they were 12 to 16 weeks old. It was observed that exposure to phthalates in adolescence promoted a decrease in anxious-like behavior in both males and females in the EPM (95). Moreover, postnatal (PND 1 to PND 30, 45, or 60) exposure to DEHP (30 mg/kg/body weight/day) induces anxiogenic-like effects in 45- and 60-day-old male rats, as demonstrated in the EPM, where the animals showed a significant decrease in entry and percentage of stay in open arms, as well as an increase in length of stay in closed arms (96). Physical exercise seems to attenuate the effects of DEHP on anxiety-like behavior. For example, rats exposed to 10 mg/kg/day of DEHP during lactation (PND 2-21) were given exercise or not (controls) for 5 weeks after weaning. Interestingly, in the EPM, animals exposed to DEHP had a reduction in the percent of entries and time spent in the open arms compared to the control group, thus demonstrating that exercise can prevent anxiogenic behavior induced by DEHP (97).

Moreover, DEHP given perinatally (GD 6 to PND 21) at relatively high doses (5, 15, 45, 135, and 405 mg/kg/day) also reduces the mounting latency in adulthood (PND 130) (98). Moreover, elevated doses of DEHP P (1500 mg/kg/day) reduce the number of ejaculations and mountings in male offspring (PND 77) exposed perinatally (GD13 to PND 21) (99). Regarding locomotor activity and working memory, it has been shown that when female rats were exposed during pregnancy (GD 14-24) to DEHP (10 mg/kg/day), their male offspring (PND 57-66) showed no changes in locomotor activity but displayed working memory deficits (100).

In a recent study, Zhao et al. (101) investigated the deleterious effects of DEHP (200 mg/kg/day) in offspring exposed prenatally in the early (GD 6 to GD 12) compared to late (GD 13 to GD 17) gestational period. The offspring were tested from PND 42 to PND 56. There were no differences between DEHP-exposed and control groups in the Open-field and EPM tests. Interestingly, in the Morris Water Maze test, the male offspring exposed to DEHP in the late gestational period display impaired spatial memory ability when compared to female offspring of the same group. Lee et al. (102) investigated the effect of DEHP on social behavior. Female mice received DEHP at a dose of 30 mg/kg/day through intragastric gavage for 4 weeks before pregnancy and also received the same dose during the gestational and lactational period (GD 0 to PND 21). Offspring were evaluated on the behavioral tests during adulthood, starting on PND 56. In the Open-field, EPM and Morris Water Maze tests, the offspring did not show differences between the experimental groups, but in the social interaction test, maternal DEHP exposure decreased offspring social preference for an intruder.

One study investigated the behavior of offspring of mice after exposure to DEHP (10, 50, and 200 mg/kg/d) from GD 7 to PND 21. In the Open-field, females exposed to 50 mg/kg/day had more anxiety-like behavior at 6 weeks of age, and females exposed to 10 and 200 mg/kg/day had more anxiety-like behavior in week 12. Additionally, females dosed with 10 and 200 mg/kg/day had a decrease in the number of rearings at week 6 compared to the control. Females at doses 10 and 50 had an increase in grooming compared to males of the same group, and males at dose 200 had an increase in grooming compared to the control, all in the 6th week. In the 12th week, females at dose 10 had an increase in the number of grooming compared to control and males from the same group. Females at doses 50 and 200 remained less time in the central area than males from the same group. In the Morris Water Maze, 6-week-old males exposed to 50 and 200 mg/kg/day of DEHP had a longer search time to identify the hidden platform and reduced the time spent in the target quadrant compared to control animals. These data suggest that perinatal exposure to DEHP affects behavior in a sex-specific manner, including locomotion activity and memory (103). Thus, these DEHP exposure studies on anxious behaviors, cognitive function, and gender differences reveals complex interactions between dosage, timing of exposure, and sex. These findings suggest that both perinatal and adolescent exposure can impact anxiety and cognition, with male and female animals responding differently to similar dosages and exposure windows.

##### 2.2.2.2 DiPeP and DBP

Pregnant rat dams were exposed to diisopentyl phthalate (DiPeP) (1, 10, or 100 mg/kg/day-1) during a period of gestation and lactation (GD 10-PNP 21). The male offspring were examined for prepubescent, pubescent, and adult behaviors in the EPM, Play Behavior, Partner Preference and Sexual Behavior Tests. There was no effect of DiPeP in the EPM or play behavior task, but in the partner preference task, the animals exposed to 1 or 10 mg/kg/day did not display any preference between male and female animals. Interestingly, the lower dose elicited greater preference for the male compartment when compared to control in the partner preference test, while in the sexual behavior test, the animals showed an increase in mount latency and intromission latency (104). In addition, another study using a mix of phthalates that included DEHP, diisononyl phthalate (DINP), and dibutyl phthalate (DBP) during pregnancy and lactation (GD 15-PND 4) at a dose of 4.5 mg/kg/day showed females had a decrease in socio-cohesive interactions in weaning (PND 21-23), puberty (PND 42-44), and adulthood (PND 77-79), while males showed a decrease only at weaning (105).

Rat dams received a diet that contained one out of the three following treatments: 20, 200, 2000, or 10000 ppm of DBP; 40, 400, 4000, or 20000 ppm of DINP; and 480, 2400, or 12000 ppm of di-(2-ethylhexyl) adipate (DEHA) from GD 15 to PND 21, and sexual behavior was evaluated in male offspring on PND 140. The number of intromissions and mounts was reduced in the males that received 40 ppm of DINP and 480 ppm of DEHA. The number of ejaculations was reduced in the DBP (200 and 2000 ppm), DINP (40 ppm), and DEHA (480 and 12000 ppm) groups. Lastly, the group exposed to 10000 ppm DBP also showed a reduced post-ejaculation interval (102). Recently, Hunter et al. (106) gave 500 mg/kg of DBP to pregnant rats. The DBP was orally administered every second day from GD14 to PND6. The sexual behavior was evaluated on the PND 60 in males and females. DBP exposure had an increase in the latency to mount compared to control groups.

Additionally, the effects on memory-related behaviors in pups of rodents that were exposed to DBP (0, 0.037, 0.111, 0.333, and 1% in the diet) were investigated from GD 6 to PND 28. The Morris Water Maze on PND 35 was used to assess spatial memory acquisition in the hidden platform test and memory retention using the probing test. Exposure to DBP influenced the spatial learning and memory of male offspring, but not in the female. The male offspring exposed to 0.037% of DBP took longer to find the escape platform, and male pups exposed to 0.037% and 0.111% of DBP had poorer memory, as they spent less time in the maze quadrant where the platform was located during the training tests. However, exposure to DBP did not alter spontaneous behavior in the Open-field in either sex or at any dose (107). Inversely, DINP administered perinatally (GD 7 to PND 17) did not induce any alteration in the spatial memory in offspring of both sexes at PND 150 as evaluated in the Radial Arm Maze Test (108).Maternal behavior and the neurodevelopment score of the offspring were evaluated in response to perinatal (GD 13-PND 15) exposure to DBP (50 and 100 mg/kg/day) using the Negative Geotaxy, and Cliff Avoidance tests in PND 7 and the Forced Swim Test and Olfactory Orientation in PND 14. It was observed that the mother mice treated with DBP had indifferent maternal misbehavior, poor nesting and recovery methods, and their offspring of both sexes showed significantly decreased scores in Negative Geotaxy, Olfactory testing and Swimming scores. However, cliff evasion decreased only in male offspring (109). Lastly, another study investigated the indirect behavioral effects of the perinatal exposure to benzyl butyl phthalate (BBP) in offspring of pregnant and lactating mothers (GD 14 to PND 23) treated with BBP (10.0 μg/ml) and examined the offspring on fear conditioning. They observed that freezing was significantly decreased in the tonal phase in males and females, and a decrease in the intertone interval was observed only in females, demonstrating learning and memory deficiencies (110).

### 2.3 Vinclozolin exposure

Vinclozolin (3-(3,5-dichlorophenyl)-5-ethenyl-5-methyl-2,4-oxazolidinedione) (VIN) is a widely used non-systemic dicarboximide fungicide that inhibits spore germination and could be used on grapes, strawberries, vegetables, fruit, ornamental plants, and turf. It is found in soil, water, and air, and thus, humans can be exposed to it through a variety of routes (111–115). Vinclozolin residues and their primary metabolites can be detectable in many natural water bodies worldwide (actual detected residue level ranges from 0.1 to 2.4 μg/L, with a maximal level being estimated to achieve 52 μg/L (116–118). The U.S. EPA estimated VIN exposure to be 2.933 μg per kg a day for the general people (119). FAO/WHO suggests the SL of VIN is set at 4 mg/kg/day, whereas acceptable TDI is approximately 0.005 mg/kg/day (120) (**Table 1**).

Intergenerational behavioral data of the VIN are shown in **Supplementary Table S3 (Supplementary material)**.

#### 2.3.1 Main mechanism actions of VIN

VIN and its active metabolites (2-((3,5-dichlorophenyl)-carbamoyl)oxy)-2-methyl-3-butenoic acid (M1) and 3¢,5¢-dichloro-2-hydroxy-2-methylbut-3-enanilide (M2) belong to a family of endocrine-disrupting compounds acting principally by competitively binding to the androgen receptor, thereby antagonizing the binding of natural androgens to the receptor, changing androgen related pathways (111, 112) (**Figure 2**). Their effective metabolite antagonized the binding of dihydrotestosterone to the rat androgen receptor *in vitro* (111). VIN downregulated the expression of many genes related to proper steroid production and development (**Figure 2**), including luteinizing hormone/chorionic gonadotropin receptor (Lhcgr), cholesterol side-chain cleavage enzyme (Cyp11a1), hydroxy-delta-5-steroid dehydrogenase (Hsd3b1), 17-beta hydroxysteroid dehydrogenase 3 (Hsd17b3), steroidogenic factor 1 (Nr5a1), platelet-derived growth factor receptor alpha (Pdgfa), anti-Mullerian hormone (Amh), desert hedgehog signaling molecule (Dhh), and SRY (sex determining region Y)-box 9 (Sox9) (121).

#### 2.3.2 Perinatal vinclozolin exposure and the impact on maternal and offspring behaviors

VIN exposure altered gene expression and urogenital differentiation in rats (111) and resulted in altered serum hormone levels, spermatogenic defects, and sexual dysfunction in rabbits (122). Neuroendocrine systems in rabbits exposed to VIN perinatally exhibited a region-specific decrease in the number of neurons immunoreactive for gonadotrophin-releasing hormone (GnRH) selectively in the region of the organum vasculosum of the lamina terminalis (OVLT) (40) and significantly increased calbindin expression in the ventral preoptic/anterior hypothalamic area (POA/AH) in both sexes of rabbit (37). Studies in rats have shown that VIN altered sexual differentiation and play behavior in male rats (123, 124), and recent work has shown transgenerational endocrine disruption of brain and behavior in third-generation descendants of rats that had been exposed neonatally to VIN with changes in amygdala and hippocampus gene expression (38, 39).

In males, exposure to 5 days of VIN (150 mg/kg body weight/day) during adulthood has been shown to induce a number of gene expression alterations in the brain as well as serum hormone changes associated with male reproductive function. For instance, it increases plasma levels of luteinizing hormone and testosterone and decreases plasma levels of thyroid-stimulating hormone and thyroxine. On the other hand, it affects the hypothalamic expression of both nuclear estrogen receptors (Esr1, Esr2) and androgen receptors, as well as in extrahypothalamic areas, such as the pituitary, striatum, and hippocampus. Lastly, VIN increases androgen receptor gene expression in the epididymis, seminal vesicle, and ventral prostate, indicating a mixed androgen receptor antagonistic/estrogen receptor agonistic action (125). Developmental exposure to VIN has been shown to induce more severe consequences in the male reproductive tract, such as testicular dysgenesis and deteriorating seminal quality, as demonstrated in rabbits perinatally exposed to VIN from GD15 to PND 4 (122).

Regarding sexual behavior, prenatal exposure (GD 8-18) to 1 mg/kg/day of VIN impairs mate preference behavior at PND 60. However, in the Odor Preference Test, odor discrimination ability was not altered by VIN (126). Further, the same group demonstrated that adult male offspring exposed prenatally to VIN have impaired sexual behavior in the sociosexual preference test. Moreover, rats exposed prenatally to VIN emitted fewer ultrasonic vocalizations (USVs) in the lactational period. During adulthood, these rats displayed an increase in intromission frequency and lordosis quotient (127). In another study using the same experimental paradigm, the same group demonstrates that F2 offspring did not show differences in USVs in the lactation period but demonstrated an increase in the sexual behavior in adulthood (128).

Interestingly, exposure to VIN during gestational and lactational periods does not seem to affect maternal behavior in rats. However, when their offspring continue to be exposed until PND 77, the females are less active in the running wheel, and there is an increased consumption of saccharin-flavored solution in adulthood in both sexes (129). Additionally, male offspring on PND 25, perinatally exposed to 1 mg/kg/day of VIN, consumed a higher volume of saccharin solution compared to the control group in the sweet preference test. The overconsumption of saccharin was compensated by lower water drinking in all other treated groups (130). Regarding social behavior, perinatal exposure to VIN from GD 14 to PND 3 has no effects on play behavior on PND 22; however, at the highest dose used (12 mg/kg), VIN increased play behavior on PND 34 in the male offspring compared to the controls (131). Finally, pregnant rats were gavaged with a daily oral dose of 0, 1.5, 3, 6, or 12 mg/kg/day of VIN from GD 14 through PND 3. The offspring were tested on PND 60 to run through a short alleyway for food reinforcement. VIN did not affect the acquisition of the response, but male offspring required more trials than females. Additionally, the lowest dose (1.5 mg/kg/day) of VIN appeared to facilitate the response in male and female offspring (132). Thus, VIN exposure produces substantial alterations in gene expression, hormone levels, and behavior, with more severe effects seen in males and those exposed perinatally. The impacts are dose-dependent and vary based on the timing of exposure, suggesting that both the developmental stage and gender play critical roles in determining the outcomes of VIN exposure. Therefore, these studies reported that bisphenols (main BPA), phthalates (main DEHP) and VIN exposure are associated with important maternal and offspring behavior abnormalities.

## 3 Transgenerational effects of EDCs

### 3.1 BPA transgenerational effects on brain and behavior

Wolstenholme et al. (133, 134) showed important effects related to transgenerational alterations in genes and behavior in a model that employed chow supplemented with 5 mg BPA/kg diet given *ad libitum* to female mice (F0 generation) 7–10 days prior to mating until the birth of pups. The behavioral analysis was performed at PND 21 in the F1 to F4 generations. Male and female mice from the F2 and F4 generations exhibited an increase in social interactions, while F1 exhibited a decrease in the Social Interaction Task, without significant effects related to anxiety-like behavior observed in the EPM. Brains from embryos (embryonic day 18.5) exposed to BPA had lower gene transcript levels for estrogen receptors (ESR1) (in male and female F1), arginine-vasopressin (AVP) (in male and female F1/F4), and oxytocin (Oxt) (only in male F4); increased levels in estrogen-related receptor γ (Esrrg) and protein-coupled estrogen receptor (Gper) (both only in male F1), demonstrating long-lasting transgenerational effects that could influence analyzed behavior. Male and female (F1) demonstrated an increase in social investigation, while males and females from the F3 generation showed a decrease in response to a novel stimulus evaluated by the Social Recognition Task, with an increase in locomotor activity in the Open-Field Test and normal olfactory discrimination response in the Odor Discrimination Task. Hyperactivity evidenced by behavioral changes detected in the Open-Field Test was not related to anxiety-like behavior, since male and female mice exposed or not to BPA transgenerationally did not differ in anxiety measures in the EPM Test.

Goldsby et al. (135), employing the same model of BPA exposure (38, 124) and analyzing brains from male and female F1 and F3 mice at PND 90, showed changes in AVP and ERα immunoreactive cells (ir) in several sexually dimorphic regions, which are part of the “social behavior network,” and could contribute to exploring mechanisms to behavioral changes anteriorly related, despite differences in the age at which analyses were performed. BPA exposure *in utero* reduced ERα-ir cell counts in the ventromedial hypothalamus (VMH) of the F1 females. Estradiol plasma levels in F3 females remained unaltered, while in the F3 brains, ERα-ir cell counts decreased in the bed nucleus of the stria terminalis (BNST), which contains primarily AVP-containing fibers, while in the anteroventral periventricular nucleus (AVPV) had more ERα-ir cells, suggesting cell-specific interactions whereby other factors (co-activators or co-repressors, for example) changed the responses of ERα.

In a new paradigm, Wolstenholme et al. (136) employed a second generation of mice bred in one out of four mating combinations that used males and females exposed and non-exposed to BPA to reveal whether the abovementioned characteristics seen in F3 offspring were acquired via maternal or paternal exposures. An effect attributed to parental lineage was specifically related to social behavior, since animals from both parents exposed to BPA transgenerationally were more active and took longer to habituate than mice from different combinations of parental lineages in the Sociability Test. Moreover, in the dishabituation trial, mice from BPA-exposed dams failed to show an increase in the investigation toward a novel mouse. Therefore, this study demonstrated that hyper-investigation during habituation was restricted to F3 mice with both parents from a BPA ancestry, and the lack of dishabituation noted in F3 BPA-lineage mice was caused by maternal BPA transmission. Although animals were not tested in the Open-Field Test in this study, previous results showed increased locomotor activity in the F3 BPA-lineage offspring, which explains the hyperactivity again registered, and also this effect of less interaction with a novel mouse could be related to already decreased response to a novel stimulus in the Social Recognition Task, even without impairment in the olfactory system (134). However, F2 and F4 generations (males and females) from BPA-exposed dams had demonstrated a significant increase in social interactions in the Social Interaction Task (133).

Results in chronological order, beginning with analysis from ED 18.5 until PND 27 neural tissues, showed that transgenerational exposure to BPA produced significant differences in several genes that are functionally and structurally related to excitatory postsynaptic synapses, such as Shank1, Homer1c, Gkap, and Psd95 (136). In the three ages examined (ED 18.5, PND 0, and PND 27) and in both regions (hypothalamus and lateral septum), BPA-lineage F3 C57BL/6J mice had expression levels of these genes that were different from those of controls: ED 18.5 BPA-lineage mice had less gene expression than controls (Shank1), and on PND 0 they had higher mRNA (Shank1 and Psd95); on PND 27, BPA-lineage brains again had less Shank1 and Psd95 expressions. Moreover, this study developed an animal model employing Friend virus B (FVB) female mice in an experimental design that used three BPA doses (0.5, 20, or 50 µg/kg/day), orally, from GD 11 until birth, generating F1 females that were used to generate F2 females, and F2 females that were used to generate F3 offspring. In these experiments, control and exposed F1 and F2 females were mated with fertility-confirmed, non-BPA-exposed males to produce the next generation to analyze the maternal inheritance. In whole brains, from FVB F3 generation male pups at PND 4, there was an increase in gene transcripts for Shank1, Homer1c, and Psd95. Authors hypothesized that the epigenetic modifications produced by BPA directly were propagated over generations and could contribute to the increments in social behavior abnormalities and disorders in humans. Transgenerational behavioral data of the BPA are shown in **Supplementary Table S4 (Supplementary material)**. Thus, these studies highlight the complex, transgenerational effects of BPA exposure, showing that both dosage and exposure window (from prenatal to postnatal stages) influence behavioral outcomes. Gender differences were also apparent, with distinct neural and behavioral changes observed in male and female offspring. The studies underscore the potential for BPA to induce long-lasting genetic and behavioral alterations, particularly related to social behavior, across multiple generations.

### 3.2 Phthalates (DEHP) transgenerational effects on brain and behavior

Brehm et al. (137) showed that prenatal exposure to DEHP had multigenerational and transgenerational effects on female reproduction in a model that orally exposed pregnant female mice to DEHP (20 μg/kg/day, 200 μg/kg/day, 500 mg/kg/day, and 750 mg/kg/day) from GD 11 until delivery. It’s important to note that changes in the estrous cyclicity, follicle numbers, and serum sex steroid hormone levels occurred despite different doses used, with effects observed in the lowest doses used in the microgram range and the highest doses in the milligram range. Estrous cyclicity was changed in the F1 (750 mg/kg/day group in all phases) and F3 generations (20 μg/kg/day, 200 μg/kg/day, and 500 mg/kg/day groups in the proestrus; 20 μg/kg/day and 500 mg/kg/day groups in the estrus; and 200 μg/kg/day, 500 mg/kg/day, and 750 mg/kg/day in the metestrus/diestrus) evaluated at 1 year of age. Evaluation of the follicle number was altered in the F1 (decreased in the primordial follicles at the 750 mg/kg/day group and in the preantral follicles at the 20 μg/kg/day group), F2 (increased in the primordial follicles at the 500 mg/kg/day group and in the primary follicles at the 200 μg/kg/day group), and in the F3 generation (increased in the primordial follicles at the 200 μg/kg/day group). The F1 generation also had a higher percentage of cystic ovaries. Testosterone serum levels were changed in all three generations, exhibiting a significant decrease (500 mg/kg/day group in the F1, 20 μg/kg/day group in the F2, and 20 μg/kg/day and 500 mg/kg/day groups in the F3); estradiol levels were changed in the F1 (increase in the 500 mg/kg/day and 750 mg/kg/day groups) and F3 (increase in the 20 μg/kg/day group), while progesterone concentrations were only changed in the F2 (decrease in the 200 μg/kg/day group).

Using a very similar exposure model to DEHP (20 μg/kg/day, 200 μg/kg/day, 500 mg/kg/day, and 750 mg/kg/day) by oral route in female mice from the F0 generation from GD 10.5 until the birth of the pups (GD 19.5), and behavioral analysis in the PND 90–100 of the F3 generation, Hatcher et al. (138) demonstrated alterations in the anxiety-like behavior and neural gene expression in both male and female mice. Male mice did not exhibit significant effects related to anxiety in the EPM, but molecular analysis showed up-regulation of estrogen receptor 2 (ESR2; 20 and 200 μg/kg/day groups) and dopamine receptor 1 (20 μg/kg/day and 750 mg/kg/day groups) in the amygdala but not in the hippocampus. However, female mice (750 mg/kg/day group) exhibited an increase in total time spent in the open arms, demonstrating an anxiolytic-related effect in the EPM. Moreover, molecular analysis in the amygdala from female mice showed down-regulation in mRNA expression of ESR1 (200 μg/kg/day and 500 mg/kg/day groups), mineralocorticoid receptor (200 μg/kg/day group), and dopamine receptor 2 (20 μg/kg/day and 750 mg/kg/day groups), while the hippocampus remained unaltered, similar to males, suggesting a brain region-specific effect of DEHP on target genes.

Previous results from Quinnies et al. (139, 140) in different models of exposure to DEHP have demonstrated alterations related to anxiety-like and social interaction behaviors, especially in males. Quinnies et al. (139) used a model that employed 150 or 200 mg/kg of DEHP by gavage to F0 generation dams between GD 7 and 14, and behavioral analysis was performed at PND 25-32 (150 mg/kg group) and PND 35-42 (200 mg/kg group) of the F3 generation. Male mice from the 200 mg/kg DEHP lineage spent more time digging and less time self-grooming, two specific nonsocial behaviors associated with an autism-like phenotype. In this model, anxiety-like behavior was not altered in any treatment or sex. However, alterations were detected in the HPA axis in two conditions: baseline and stressed by chronic restraint stress (CRS). In baseline conditions, F3 females exposed to DEHP (150 mg/kg) had higher serum corticosterone levels and lower corticotropin-releasing hormone receptor 1 (Crhr1) mRNA expression in the pituitary, which also was observed in F3 males but not accompanied by changes in serum corticosterone levels; and in the stressed conditions, F3 females had lower serum corticosterone levels and higher Gnas (guanyl nucleotide-binding protein) mRNA expression in the pituitary, which also was observed in F3 males exposed, again without changes in corticosterone levels.

Quinnies et al. (140) employed a model of exposure to DEHP from GD 0 until the lactation day 10. The dams consumed a cocoa puff coated in 50 μL of stripped corn oil containing doses of DEHP equivalent to 0.5 (low dose), 40 (medium dose), or 400 (high dose) μg/kg body weight per day, and maternal and offspring behaviors were observed. Analysis of DEHP metabolites demonstrated that these doses generated the range of levels already measured in human blood since serum concentrations of MEHP for 400 μg/kg dosed dams averaged 160 ± 17.2 ng/ml and 1.6 ± 0.4 ng/mL for 5-OH-MEHP. Metabolite concentrations of pooled embryo serum from 400 μg/kg-dosed dams averaged 96.7 ± 36.9 ng/ml for MEHP and 1 ± 0.20 ng/ml for 5-OH-MEHP. Maternal behavior was analyzed on PND 2, 4, and 6 in the F0 and F2 generations, demonstrating that F0 dams did not display any DEHP-related differences in maternal care, but as expected, the age of the litter significantly affected two measures: time in nest and eating. The direction of the differences was consistent with dams spending more time in the nest with younger (PND 2) versus older (PND 6) pups. In the same way, F2 dams did not show any effects of DEHP dose lineage, but spent more time licking and grooming their litters on PND 2 than on PND 4. Moreover, F0 dams spent less time in the nest licking and grooming pups and nursing and spent more time digging in the cage as compared with the F2 dams. Analysis of the male and female F1 offspring from the 40 and 400 μg/kg exposed groups showed a reduction in the socially investigative behaviors and an increase in the exploration behavior (Social Interaction Test), while F1 offspring from the 0.5 and 40 μg/kg exposed groups exhibited increased anxiety-like behavior (EPM). In the male F3 offspring from the 400 μg/kg-exposed group, there was an increase in the investigative behaviors, and the increase in the anxiety-like behavior was accentuated. Female F3 offspring did not exhibit changes related to anxiety but also had impairments in the social behaviors. Transgenerational behavioral data of the phthalates (DEHP) are shown in **Supplementary Table S5 (Supplementary material)**. Overall, these studies underline the complex interactions between dosage, exposure windows, and gender, revealing the potential for DEHP to cause significant multigenerational and transgenerational effects. These findings are especially relevant for understanding how even low-dose exposures to environmental chemicals during pregnancy can affect not just the direct offspring but also future generations.

### 3.3 VIN transgenerational effects on brain and behavior

VIN was the first environmental EDC shown to have transgenerational effects and to cause dependent sex- and age-specific effects on disease induction, mate preference, locomotor, social, and anxiety-related behavior, changes in the brain transcriptome, and sperm epigenome (38, 39, 141–146). A simultaneous study (147) employing pregnant dams exposed to VIN by oral gavage (10 mg/kg and 50 mg/kg) from GD 13 through 17 and assessed the fetal genital tubercles from exposed fetuses on GD 19 demonstrated that VIN in the relatively low dose caused hypospadias in the mice, but it also virilized the females, suggesting direct or indirect effects on progesterone receptor expression. Transgenerational behavioral data of the VIN are shown in **Supplementary Table S6 (Supplementary material)**.

Employing an experimental model that consisted of administering a 100 mg VIN/kg of weight daily by IP injections from GD 8 until 14 to female rats in the F0 generation, Crews et al. (38) showed that epigenetic transgenerational inheritance of EDCs action could represent an unappreciated force in sexual selection. Females three generations removed from the exposure discriminate and prefer males, who do not have a history of exposure, in the Mate-Preference Behavioral Analysis and Odor-Salience Analysis. However, males epigenetically imprinted do not exhibit such a preference in the Mate-Preference Behavioral Analysis but spend more time exploring non-VIN-exposed females in the Odor-Salience Analysis.

Skinner et al. (39) showed opposite effects in relation to anxiety-related behavior, since VIN-exposed males had a decrease in anxiety-like behavior, while the females had an increase in anxiety-like behavior. The age of animals also influenced the observed effect because males exhibited anxiety-like behavior represented by an increase in the time spent on the light side of the box and in the number of transitions between the light and dark sides of the box (Light: Dark Box) and an increase in the number of total arm entries (EPM) in the “young” age set of analysis (PND 82-155), while females tended to have a shorter latency to enter the dark side and a significant decrease in time spent in the light side of the box, with no differences in the number of transitions (Light: Dark Box) in the “aged” age set of analysis (PND 369-386). In the EPM, female “aged” and “young” (PND 93-124) showed a decrease in both the percent time spent on the open arms and in the percent of open arm entries, with no difference in the number of total arm entries, confirming the anxiety-like behavior already in “young” age.

Brains from males and females were collected to analyze transgenerational alterations in the hippocampus and amygdala transcriptomes (39). Male brains were collected at 12 months of age and females at 15 months of age, and results demonstrated that VIN promotes a sex-specific transgenerational programming of the brain transcriptomes. In the males, the expression of 92 genes in the hippocampus and 276 genes in the amygdala were transgenerationally altered, while in the females, the expression of 1301 genes in the hippocampus and 172 genes in the amygdala were transgenerationally altered. Analysis of specific gene sets demonstrated that several brain signaling pathways were influenced, including those involved in transcriptional regulation, signal transduction, cytoskeleton, metabolism, cell cycle, development, proteolysis, and apoptosis. As previously demonstrated by Anway et al. (141, 142), VIN-exposed F1-F4 generations had a higher frequency of transgenerational disease induction, since animals greater than 6 months developed various diseases such as prostate disease, kidney disease, immune abnormalities, and tumor development but had no major effects on hormone levels (progesterone, estradiol, testosterone, and corticosterone).

Employing the same model of VIN exposure (38, 39) and additionally exposing male adolescent rats to stress (143), it was hypothesized that epigenetic reprogramming of the brain regions could alter the gene networks and pathways identified to promote the altered possible behavioral phenotypes. They observed that chronic restraint stress (CRS) during adolescence (PND 26-46) in the VIN-exposed transgenerationally male rats had no effects related to depression-like behavior but promoted increased anxiety-like behavior in the Open-Field Test, represented by more movements from the center into corners, evaluated in adulthood (PND 114-118). In general, stressed males moved faster through the center than non-stressed males, indicating that CRS increased anxiety later in adulthood. Moreover, in the Social Tests, non-stress VIN-exposed males visited the stimulus animal for longer periods and moved more between chambers, while VIN-exposed males again showed effects of stress, traveling farther and faster than VIN non-stress males and also spending less time in the center compartment and more time with the familiar and novel stimulus males. This proposed “two-hit” model, where the “hits” span generations, in this instance the first hit (transgenerational epigenetic inheritance by VIN exposure in the F0 female) predisposing a future generation to respond to a second hit (CRS during adolescence), which further alters the adult phenotype, was accompanied by changes in body weight, gonadosomatic/adrenosomatic indices, corticosterone/testosterone levels, brain metabolism/genomics, and gene networks. Neural areas analyzed included CA1 and CA3 of the hippocampus, the basolateral amygdaloid nucleus (BLA), and the primary and secondary motor cortex (CRTX). The altered genes were related to cellular functions and processes such as receptors and binding proteins, metabolism, transcription, signal transduction, and development. Interestingly, the highest correlated pathway was olfactory transduction, with 78 genes altered, and other brain-related pathways affected by VIN exposure and stress were neuroactive ligand-receptor interaction, Huntington disease, Alzheimer’s disease, axon guidance, and Parkinson disease. Two of the more ubiquitous pathways affected were the calcium signaling pathway and the MAPK signaling pathway.

Similarly, Gillette et al. (144) behaviorally characterized male and female F3 VIN- or vehicle-lineage rats, stressed or non-stressed. CRS applied during adolescence (PND 23-44) and behavioral testing during adulthood (PND 90-118) demonstrated that male rats exposed to CRS in adolescence showed decreased anxiety behaviors in adulthood, evaluated by Open-Field Test and Light: Dark Box, while female rats did not show any differences caused by CRS. In adulthood, male rats presented a higher preference to associate with an animal as opposed to an empty cage and had no preference for social novelty, while female rats also presented a higher preference to associate with an animal as opposed to an empty cage, but they spent more time with a novel animal as opposed to a familiar one, being all analysis obtained in the Sociability and Social Novelty Test. These results suggested that males and females differ in the compensatory strategies acquired with early life stress. Cytochrome oxidase histochemistry, a measure of brain metabolic activity, was measured in neural nuclei, and a significant increase in metabolic profile due to stress and VIN exposure was detected in females, while males had a decrease of lesser magnitude. Sex-specific effects were also found in the gene expression of specific brain regions, since females expressed changes in the hippocampus, specifically in the CA3 region, demonstrating an up-regulation by VIN of the ESR1 and ESR2 that mediates effects of estradiol on neuronal activity; Crhr1 and Drd2 receptors, involved in cognitive function; and stress reactivity growth factors Ptgds and Tgfa. In turn, males expressed changes in the amygdala, more specifically in the basolateral amygdaloid nucleus, a region involved in modulating fear and anxiety responses, which had upregulated Bdnf and Negr1 gene expression.

Using the same model of transgenerational exposure to VIN, Nilsson et al. (145) analyzed the sperm epigenome of F3 generation rats with specific abnormalities to find epigenetic biomarkers of transgenerational disease. Behavioral analysis performed at 11 months of age in the female F3 generation VIN-lineage rats showed no locomotor or anxiety-related changes, but male F3 generation VIN-lineage rats spent significantly less time in the closed arm of the EPM, and there was a trend toward them spending more time in the open arm, exhibiting an increase in locomotor activity. In relation to disease or abnormalities observed, F3 generation VIN lineage rats had increased disease rates in the testis, prostate, kidney, and ovary, demonstrating transgenerational inheritance of increased susceptibility to any of these diseases alone or simultaneously. In addition, a sex-specific effect observed in the female F3 generation VIN-lineage rats at 12 months of age was a significant transgenerational increase in obesity rates, evaluated by adipocyte size in the gonadal fat pad, as well as body mass index. Epimutation signatures were distinct between all the related disease conditions, suggesting that possibly epigenetic biomarkers in the sperm may provide a useful molecular diagnostic and could have a significant impact on diagnosis and management (145).

A recent mechanism that was explored in relation to VIN transgenerational effects (146) employed an exposure model that consisted in administering 1 mg VIN/kg of weight by daily intraperitoneal injection to F0 pregnant dams from GD 8 to GD 18, with F1 and F3 male rats euthanized at PND 120. It is important to note that authors used a breeding paradigm in which F1-treated males mated with untreated females to yield the F2 generation. This was repeated in the F2 generation, resulting in F3 males that derived from paternal transmission of the germline. Sperm and brains were analyzed to evaluate direct and transgenerational effects of low-dose VIN on methylated regions (DMRs) across CpG islands, regions with a higher-than-expected density of CG dinucleotides, across the genome. In the sperm, 74% of the differentially methylated CpG islands due to ancestral low-dose VIN exposure were hypermethylated, which can compromise the integrity of the germline, since CpG islands in sperm have very low levels of methylation, presumably to protect against spontaneous deamination and subsequent mutation. In the brain nuclei, more specifically, in the CeA and CA3 of the hippocampus, VIN exposure showed an equivalent number of DNA methylation alterations between generations. As CeA integrates sensory information to initiate physiological and behavioral output and the CA3 of the hippocampus modulates fear and anxiety responses, behavioral changes demonstrated anteriorly (38, 39, 147) can be related to epimutations that contribute to the phenotype of brain disorder caused by transgenerational exposure to VIN.

A critical factor to discuss is related to the doses used, since behavioral changes were demonstrated in studies that employed 100 mg/kg b.w. daily by intraperitoneal injections from GD 8 to GD 14 (38, 39, 143, 144), while Gillette et al. (146) treated animals with 1 mg/kg/day from GD 8 to GD 18, a dose that is lower than the no observed adverse effect level for VIN (VIN NOAEL (chronic dietary) = 1.2 mg/kg/day). Interestingly, Krishnan et al. (148) demonstrated behavioral changes with an exposure model that evaluated maternal behavior of the F2 females towards their F3 generational offspring and F3 physiological and behavioral outcomes. In this exposure model, the F0 dams received a daily intraperitoneal injection of 1 mg VIN/kg/day from GD 8 to GD 18. Males and females from the F1 generation were bred with untreated stimulus animals to form the paternal and maternal lineages of the F2 generation, and adult paternal males and maternal females from the F2 generation were bred with untreated mates to produce the F3 generation. Maternal behavior was evaluated in the maternal lineage F2 dams on PND 3 during the light-on phase and showed an increase in the numbers of licking and grooming events caused by VIN exposure. Observations in the dark phase, in the maternal lineage F2 dams, showed an increase in numbers of bouts on the nest, and in the paternal lineage F2 dams, an increase in numbers of nursing bouts and active nursing positions. Maternal lineage pups in the F3 generation were evaluated on PND 3 and PND 6, and they had a decrease in USV call durations, while paternal lineage pups in the F3 generation exhibited no changes. In adults (PND 75-100), males from maternal lineage F3 moved more quickly but spent less time immobile, suggesting lower levels of stress reactivity, while females moved more quickly and spent less time in the center of the chamber, suggesting higher stress reactivity, evaluated by the Open-Field Test. Females from the paternal lineage F3 generation showed an increased immobility time in the Light: Dark Box. Thus, VIN exposure exemplifies how environmental factors can induce complex, sex- and age-specific transgenerational effects, including altered behaviors, epigenetic changes, and increased disease susceptibility. These studies underscore the need for further exploration of the mechanisms behind EDCs and their long-term impacts on both individual health and generational inheritance. Therefore, these studies reported that bisphenols (main BPA), phthalates (main DEHP) and VIN exposure are associated with important transgenerational behavior abnormalities.

## 4 Future perspectives

Despite growing concern regarding the neurodevelopmental impact of endocrine-disrupting chemicals (EDCs), experimental studies investigating the intergenerational and transgenerational effects of key EDCs—specifically bisphenols (BPA, BPS, BPF, and BPAF), phthalates (DBP, BBP, DEHP, and DiPeP), and vinclozolin (VIN)—on brain and behavioral development remain limited. Comprehensive data across generations are essential to accurately assess long-term risks posed to current and future human populations. A critical limitation in many existing studies is the use of exposure doses that do not reflect the concentrations detected in human biological samples, reducing translational relevance. Moreover, the majority of research has focused predominantly on maternal transmission pathways, while paternal lineage effects are comparatively underexplored. Even more rarely addressed are studies evaluating both parental lineages in parallel. Given that real-world exposures often involve complex mixtures of EDCs, there is a pressing need for studies that investigate the combined effects of multiple EDCs on neural development and behavior.

## 5 Conclusions

Bisphenols (BPA, BPS, BPF, and BPAF), phthalates (DBP, BPP, DEHP, and DiPeP), and vinclozolin (VIN) have effects on brain development and behavior across generations in experimental animals. The effects observed are dependent on maternal or paternal transmission, period of exposure, dose, sex, and period of behavioral analysis. The behavioral analyses used in the available studies detected motor alterations and changes related to anxiety, depression, and cognition, in addition to effects related to sociosexual behavior. The effects of EDCs arise from endocrine changes and also from direct actions on signaling pathways related to the transcriptional regulation of different genes involved in signal transduction, cytoskeleton formation, metabolism, cell cycle, development, proteolysis, and apoptosis. Transgenerational epigenetic inheritance, in which phenotypic alterations occur in generations unexposed to the initial environmental insult, represents a critical frontier in neurotoxicology. However, most studies to date have limited their scope to intergenerational effects, typically assessing only F1 and occasionally F2 offspring. Research exploring the persistence of EDC-induced changes in F3 or subsequent generations, particularly in the context of neurogenesis, synaptic plasticity, or behavior, remains rare. The absence of such data impedes a comprehensive risk assessment relevant to human populations. Moreover, real-life human exposure to EDCs rarely involves isolated compounds; rather, individuals are routinely exposed to complex mixtures with potentially synergistic, antagonistic, or additive effects. Yet, most experimental studies continue to evaluate single-compound exposures. There is a pressing need for studies employing mixture-based approaches, using factorial designs or environmentally representative EDC cocktails, to better capture the interactive effects of multiple EDCs on neurodevelopmental trajectories. Collectively, addressing these gaps—through the use of environmentally relevant dosing, inclusion of both parental lineages, and mixture-based exposure models—will be essential for generating ecologically valid insights into how EDCs influence brain development and behavior across generations.
