# Supplementary material for: Intergenerational and transgenerational effects of endocrine-disrupting chemicals in the offspring brain development and behavior
Source: Front Endocrinol (Lausanne). 2025 May 21;16:1571689. doi: 10.3389/fendo.2025.1571689 (PMC12133532; doi:10.3389/fendo.2025.1571689)
Supplement: Supplementary file 1 [file DataSheet1.docx]

Supplementary Material

**Intergenerational and transgenerational effects of endocrine-disrupting chemicals in the offspring brain development and behavior**

**Glaecir Roseni Mundstock Dias^1^, Fabiana Cardoso Vilela Giusti^2^; Cíntia Onofra de Novais^2^, Maria Aparecida Lima de Oliveira^2^, Alexandre Giusti Paiva^2^, Bruna Kalil-Cutti^3^, Megan M Mahoney^4^, Jones Bernardes Graceli^5,6*^**

^1^ Postgraduate Program in Endocrinology, Faculty of Medicine, Federal University of Rio de Janeiro; Precision Medicine Research Center, Carlos Chagas Filho Institute of Biophysics, Federal University of Rio de Janeiro, Rio de Janeiro, RJ, Brazil.

^2^ Institute of Biomedical Sciences, Federal University of Alfenas, Alfenas, MG, Brazil.

^3^ School of Medicine, Faculty of Science for Health, Federal University of Rondonópolis, Rondonópolis, MT, Brazil.

^4^ Comparative Biosciences, University of Illinois System, Urbana, 61802, United States.

^5^ Department of Morphology, Health Sciences Center, Federal University of Espírito Santo,Vitória, ES, Brazil.

^6^ Animal Science, School of Agricultural Sciences, Southern Illinois University, Carbondale, IL, USA.

*** Correspondence:**Jones B Graceli

[jbgraceli@siu.edu](mailto:jbgraceli@siu.edu)

# 1 Supplementary Tables

**Table S1:** EDCs effects on rodent intergenerational behavior: Bisphenols studies………………………………………….……………………………………………………………………………………………………pp.3

**Table S2:** EDCs effects on rodent intergenerational behavior: Phthalates studies…………………………………………………………………………………………………………………………………….….…….pp.18

**Table S3:** EDCs effects on rat intergenerational behavior: Vinclozolin (VIN) studies……………………………………………………………………………………………………………………………………….……..pp.27

**Table S4:** EDCs effects on mouse transgenerational behavior: BPA studies…………………………………………………………………………………………………..………………………………………….pp.30

**Table S5:** EDCs effects on mouse transgenerational behavior: DEHP studies………………………………………………………………………………………………………………………………………...……pp.32

**Table S6:** EDCs effects on rat transgenerational behavior: Vinclozolin (VIN) studies……………………………………………………………………………………………………………………………………………...pp.34

**Table S1:** EDCs effects on rodent intergenerational behavior: Bisphenols studies

| **Species** | **Dose of EDCs used and adm. route** | **Exposure period** | **Behavioral outcomes in dams** | **Time of behavioral analyses in the offspring** | **Behavioral tests performed on offspring** | **Behavioral findings found in offspring** | **Male and/or female** | **Reference** |
| --- | --- | --- | --- | --- | --- | --- | --- | --- |
| Sprague Dawley  rats | BPA 40 and 400 µg/kg  Intragastric gavage | 10 days before mating until the weaning of the pups | - | PND 85 | Holeboard  Elevated plus-maze | Duration of head dipping showed a decrease in both dosages, while  the frequency of head dipping was reduced only in high dosage and crosses were reduced with the low dosage in female.  Reduced frequency of head dipping in high dose males.  In females, decrease by the high dosage treatment on the number of closed-arm entries and the number of total entries.  In males, low-dose maternal treatment increased the number of open-arm entries, the percent of open-arm  entries and the percent of time spent in the open arms; high dosage treatment increased the percent of open-arm entries. | Both | Farabollini et al.  (1999) |
| Sprague Dawley  rats | BPA 3.2, 32 and 320 mg/kg/d  Intragastric gavage | GD 11 to PND 20 | - | 6 months | Lordosis behavior | No changes. | Females | Kwon et al. (2000) |
| Sprague Dawley rats | BPA 40 and 400 μg/kg  Intragastric gavage | PND 0-21 or GD 14 to PND 6 | - | PND 35, 45, and 55 | Social play behavior | Masculinization of female behavior in two of these categories (play with females and socio-sexual exploration) intensification of male behavior in males (play with females). | Both | Dessì-Fulgheri et al.  (2002) |
| Sprague Dawley rats | BPA 40 µg/kg/d  Intragastric gavage | Pregnancy and lactation | - | Adulthood | Intruder test  Sexual orientation  Male sexual activity  Female sexual activity | Increase in defensive behavior in males.  No changes in male.  Increase in sexual motivation in female.  Impairment of sexual performance.  Increase in receptive behavior. | Both | Farabollini et al.  (2002) |
| Sprague Dawley  rats | BPA 0.04 mg/kg  Intragastric gavage | Pregnancy and lactation | - | PND 35-45 and PND 70 | Novelty preference  Impulsivity  Open-field with amphetamine | Females spent significantly less time.  Reduced level of impulsive behavior in both.  Increment activity was significantly less marked in BPA-treated male rats. | Both | Adriani et al. (2003) |
| F344/N rats | BPA 4, 40 and 400 mg/kg/d  Intragastric gavage | GD 10 to PND 20 | - | PND 7, 21, 28, 56 and 84 | Spontaneous activity  Shuttle box avoidance  Open-field | Elongated immobile time in the dark phase in female.  Responses significantly higher at 4 weeks of age in male.  Lower responses at 8 weeks of age in male.  Higher percentage of grooming at 8 weeks of age in male. | Both | Negishi et al.(2003) |
| F344/N rats | BPA 0.1 mg/kg/d  Intragastric gavage | GD 3 to PND 20 | - | 15 weeks | Open-field  Elevated plus maze  Passive avoidance  Active avoidance | No changes.  No changes.  No changes.  Less avoidance responses to test stimuli. | Males | Negishi et al.(2004) |
| Sprague Dawley rats | BPA 40 µg/kg/d  Intragastric gavage | Pregnancy and lactation | - | PND 35-55 | Socio-sexual behavior | Decrease of play with males and social grooming. | Female | Porrini et al. (2005) |
| Wistar rats | BPA 0.01ppm  Drinking water | GD 13-PND 0 | - | 6 to 9 weeks | Open-field  Elevated plus maze  Passive avoidance  Forced swimming | Increased duration of rearing in females.  No changes.  No changes.  Increased immobility time in males. | Both | Fujimoto et al. (2006) |
| Long Evans  rats | BPA 2, 20, and 200 mg/kg/d  Intragastric gavage | GD 7 to PND 18 | - | Adulthood | Sexual behavior  Sucrose preference  Activity in maze | No changes.  No changes.  No changes. | Females | Ryan et al. (2009) |
| ICR mice | BPA  0.5, 5, and 50 mg/kg  Intragastric gavage | GD 7-PND 21 | - | PND 21 and PND 56 | Morris water maze  Step-down passive avoidance | Increased latency to find the hidden platform in 3 doses and 0.5 or 5 mg/kg/d decreased the percentage of time spent in the quadrant where the platform.  Error frequency to step down was increased and the latency of the step-down response onto the grid floor 24 hours after footshock was reduced by exposure to BPA at 5 and 50 mg/kg in the PND21 or at 50 mg/kg in the PND56. | Males | Xu et al. (2010) |
| Long Evans rats | BPA 5, 50, and 500 μg/kg bw/d, or 5 mg/ kg bw/d  Intragastric gavage | GD 7-PND 14 | - | PND 90-120 | Sexual performance in males  Sexual performane in female | At 50 μg/kg bw/d adult sexual performance impaired and improved appetitive behavior at 5 mg/kg bw/d.  No changes. | Both | Jones et al. (2011) |
| Long Evans rats | BPA 5, 50, 500, and 5000 μg/kg/d  Intragastric gavage | Pregnancy and lactation | - | PND 90-150 | Morris water maze  Forced swimming  Elevated plus maze | No changes.  Low doses (5 μg/kg) of BPA eliminated sex differences found between controls.  Low doses (5 μg/kg) of BPA eliminated sex differences found between controls. | Both | Jones and Watson (2012) |
| Wistar rats | BPA  1 mg/L  Drinking water | GD 6-PND 40 | - | PND 24-28 and PND 60-70 | Light/dark box  Elevated plus maze | Increased latency to reach light chamber and reduced permanence in light chamber in juveniles rats in both sex.  Reduction in the number of entries into open arms in juveniles rats in both sex. | Both | Patisaul et al. (2012) |
| Sprague Dawley  rats | BPA  0.4 and 4 mg/kg/d  Intragastric gavage | GD 7-20 or PND 1-14 | - | PND 56 | Open-field  Light-Dark  Mirrored maze  Elevated plus maze  Forced swimming  Open-field  Light-Dark  Mirrored maze  Elevated plus maze  Forced swimming | Gestational exposure:  Increased grooming of females in the 4 mg group.  Reduction in permanence time on the light side by males in group 0.4 mg and males and females in group 4 mg. Reduction in the number of entries on the light side by females and males in group 0.4 mg and females in group 4 mg.  The time spent in the mirror by females in group 0.4 mg was lower as well as in males in group 4 mg.  Less time in open arms for 0.4 mg and 4 mg females. Reduction in the number of entries into the open arms of both males and females 0.4 and 4 mg groups. Decrease in head dips of males and females 0.4 and 4 mg groups.  Greater immobility of males and females 0.4 and 4mg groups.  Lactation exposure:  No changes.  Decrease in permanence time on the light side for females in the 0.4 and 4 mg groups. Reduction in the number of entries on the light side by females of the 4 mg group.  Increased nº of male inputs 0.4 and 4 mg groups in the mirror chamber.  Less time in open arms for 0.4 and 4 mg female groups. Reduction in the number of entries into the open arms of 0.4 females and 4 mg males and females groups. Decrease in head dips of males and females 0.4 and 4 mg groups.  Increased immobility of males and females 4 mg groups. | Both | Xu et al. (2012) |
| Wistar rats | BPA  24 μg/kg/d  Drinking water | PND 0-PND 7 | - | 6-9 weeks | Open-field  Elevated plus maze  Forced swimming | Duration of rearings increased in males.  No changes.  Increased immobility time and reduced latency. | Both | Fujimoto et al. (2013) |
| CD-1 mice | BPA  10 μg/kg bw/d  Drinking water | GD 11-PND 8 | - | PND 28-30 and PND 70 | Novelty test  Open-field  Elevated plus maze | At both testing ages,both pre- and postnatally BPA-exposed females were less prone to explore a novel environment.  No changes.  No changes. | Both | Gioiosa et al. (2013) |
| BALB/c mice | BPA  2, 20, and 200 μg/kg/d  Intragastric gavage | GD 0-19 | Increased maternal behavior. | PND 30-70 | Home cage social behavior  Open-field  Social approach and aggression | Females sniffed more and males followed more in all doses.  200 µg females had a reduction in distance traveled and reduced time in the center and males had an increase in the center of the same dose.  Males and females had an increase in aggressive behavior at the dose of 200 µg. | Both | Kundakovic et al.  (2013) |
| Sprague Dawley  rats | BPA  50 and 500 µg/kg/d  Intragastric gavage | GD 10 to PND 14 | - | PND 49-77 | Open-field  Elevated plus maze  Maze test  Morris water maze  Step-through passive avoidance | No changes.  No changes.  BPA 50 male group needed more time to reach the reward.  No changes.  Higher latency of the BPA 50 group. | Both | Kuwahara et al.  (2013) |
| C57BL/6J mice | BPA  250 ng/kg/d  Subcutaneous injection | GD 10 to PND 20 | - | 4 and 9 weeks | Fear  conditioning | Enhanced fear memory. | Both | Matsuda et al. (2013) |
| Sprague Dawley  rats | BPA 40 mg/kg/d  Drinking water | Pregnancy and lactation | - | PND 38-45 | Open-field | Reduction in exploration time in the central area. | Females | Zhou et al. (2013) |
| CD-1 mice | BPA 50 mg/kg  In diet | 2 weeks before mating and during pregnancy and lactation | - | PND 24 | Open-field  Elevated plus-maze | BPA females spend less time in the center and travel less distance.  BPA females spend less time, enter less and travel less distance in open arms. | Both | Luo et al. (2014) |
| Long Evans  Rats | BPA 4, 40, and 400 μg/kg  Intragastric gavage | Pregnancy and the pups GD 1-9 | - | PND 85 | Radial arm maze | No changes. | Both | Sadowski et al. (2014) |
| Sprague Dawley  rats | BPA 0.05, 0.5, 5 and 50 mg/d  Intragastric gavage | GD 9-20 | - | PND 21 | Open-field  Radial arm maze task | BPA at 0.5 and 5 mg/kg showed a decrease in the number of grid crossings and rearings; BPA groups (0.05, 0.5, and 50 mg/kg) shown a slightly lower frequency of grooming.  BPA 0.05 mg group increased the mean number of errors committed. | Males | Wang et al. (2014) |
| Wistar rats | BPA  0.1ppm  Drinking water | GD 13-21 | - | 10-14 weeks | Responses to a predator odor by using a novel cross-form apparatus | Higher odor-avoidance response. | Both | Fujimoto et al. (2015) |
| Sprague Dawley  rats | BPA 40 mg/kg/d  Drinking water | Pregnancy and lactation | - | PND 40-50 | Open-field  Elevated plus maze  Light dark | Longer time spent in the central area.  Lower percentage of time and number of entries in open arms.  Less time spent on the light side of the box. | Females | Zhou et al. (2015) |
| Sprague Dawley  rats | BPA 40 µg/kg/d  Intragastric gavage | Pregnancy and lactation | - | PND 60 and PND 85 | Morris water maze  Elevated plus maze | Escape latency increased and decreased the percentage of time spent in the target quadrant.  No changes. | Both | Chang et al. (2016) |
| Wistar rats | BPA 25 µg, 250 µg, 5 mg, and 50 mg/kg bw/d  Intragastric gavage | GD 7 to PND 22 | - | 4-7 months | Elevated plus maze  Morris water maze  Sweet preference | No changes.  Altered spatial learning in female.  No changes. | Both | Hass et al. (2016) |
| Wistar rats | BPA  1 mg/L  Drinking water | Pregnancy and lactation | - | PND 24-28 and PND 60 | Social choice  Open-field | No changes.  No changes. | Both | Hicks et al. (2016) |
| Sprague Dawley  rats | BPA 2.5, 25, and 2500 μg/kg/d  Intragastric gavage | GD 6 and up to PND 21 directly on the offspring | - | PND 84 | Barnes Maze | 2500 BPA group sniffed more incorrect holes on day 7. The 2500 BPA females were less likely to locate the escape box in the allotted time whereas 2.5 BPA males showed improved latency. | Both | Johnson et al. (2016) |
| C57BL/6NCrSlc mice | BPA and BPF  10 mg/kg  Intragastric gavage | GD 11.5-18.5 | - | 10 weeks | Open-field  Elevated plus maze  Forced swimming | Lower percentage of permanence in the central region in BPA and BPF groups’ females.  Longer time spent in closed arms in males and females BPA and BPF groups.  Higher percentage of immobility time in BPF group females. | Both | Ohtani et al. (2016) |
| CD-1 mice | BPS 2 and 200 μg/kg/d  Wafer | GD 9 to PND 20 | BPS 200 group had a decrease in nest time and an increase in time to catch pups in PND 14.  No changes in Open-field test. | Adulthood | Maternal behavior  Open-field | Decrease in nest dwell time in PND2 and 7 in both doses. Increased nest building time at dose 200 in PND14.  No changes. | Females | Catanese and Vandenbeg (2017) |
| ICR mice | BPAF  0.4 and 4.0 mg/kg  Intragastric gavage | GD 1-19 | - | PND 35 | Open-field  Novelty-suppressed feeding  Sucrose preference  Tail suspension  Forced swimming  Novel object recognition  Contextual fear conditioning | Reduced latency in the central zone in males at 0.4 or 4 mg/kg.  In female, BPAF 0.4 mg/kg dose reduced the latency to feeding.  Reduction of sucrose preference in males at a dose of 4 mg/kg.  Increase in immobility time in males at both doses.  In female, BPAF 0.4 mg/kg dose increased floating time.  Maternal BPAF 0.4 or 4 mg/kg decreased the recognition index in the long-term memory in both sexes.  Maternal BPAF 0.4 or 4 mg/kg decreased the freezing time of male. | Both | Gong et al. (2017) |
| Swiss albino mice | BPA  50µg/kg/d  Intragastric gavage | GD 7 to PND 21 | - | 3 and 8 weeks | Open-field  Elevated plus maze | Reduction of the time spent, number of entries and distance traveled in the central zone in 8 weeks.  Decrease in time spent and number of entries to the open arms. | Males | Kumar and Thakur (2017) |
| CD-1 mice | BPS  2 and 200 µg//kg/d  Small wafer | GD 9-PND 20 | Dams 200 µg cared less in PND 14. Increased high crouch time in all doses. Decrease in the percentage of breastfeeding puppies at the 200 µg dose. | PND 2, 7 and 14 | Evaluation of pup-initiated nursing | Puppies of both doses were less likely to initiate breastfeeding. | Both | LaPlante et al. (2017) |
| Long Evans  rats | BPA 40 and 400 µg/kg/d  Cookie | GD 1-21 | Reduction in the number of lickings | PND 26-40 and PND 90 | Periadolescent social behavior  Elevated plus maze | Decreased social behavior in both.  No changes. | Both | Wise et al. (2018) |
| C57BL/6J mice | BPA  10 μg/kg and 10 mg/kg  In diet | Mating, gestation, and lactation | No changes. | 16-20 weeks | Hole poke  Elevated zero maze  Social choice  Forced swim | No changes.  No changes.  No changes.  Increased immobility time in males in 2 doses. | Both | Xin et al. (2018) |
| Wistar rats | BPA 10 and 50 μg/kg/d  Intragastric gavage | Pregnancy and lactation | - | PND 160 | Open-field  Elevated plus-maze | No changes.  BPA 10 females entered arms more often closed and showed a smaller number of head-dips; BPA 50 females showed only a smaller number of head-dips. | Both | Silva et al. (2019) |
| Wistar rats | BPA 25 µg/L, 250 µg/L or 2.5 mg/L  Drinking water | GD 9-21 | - | PND 21-29 | Open-field  Light dark  Elevated plus maze  Novel object recognition | Increased immobility time for males and females. Males spent less time in the center of the apparatus.  Males remained on the light side less time.  Decrease in the number of entries and time spent in open arms and longer stay in closed arms in both sexes.  No changes. | Both | Raja et al. (2020) |

Adm= administration; d= day; GD=gestational day; PND=postnatal day; (-) not analyzed; rodent= rat and/or mouse; BPA= bisphenol A; BPS= bisphenol S.

**Table S2:** EDCs effects on rodent intergenerational behavior: Phthalates studies

| **Species** | **Dose of EDCs used and adm. route** | **Exposure period** | **Behavioral outcomes in dams** | **Time of behavioral analyses in the offspring** | **Behavioral tests performed on offspring** | **Behavioral findings found in offspring*** | **Male and/or female** | **References** |
| --- | --- | --- | --- | --- | --- | --- | --- | --- |
| Sprague Dawley  rats | DEHP  0, 375, 750, and 1500 mg/kg/d  Intragastric gavage | GD 3 to PND 21 | - | PND 77 | Sexual behavior | Males at dose of 1500 mg/kg/day showed reduction mount and ejaculations. | Males | Moore et al. (2001) |
| Wistar  rats | Low doses: 0.015, 0.045, 0.135, 0.405 and 1.215 mg DEHP/kg/d  High doses: 5, 15, 45, 135 and 405 mg DEHP/kg/d  Intragastric gavage | GD 6 to PND 21 | - | PND 130 | Sexual behavior | Males at high dose of 5 and 15 mg/kg/day showed reduction mount latency. | Males | Andrade et al., (2006) |
| Wistar rats | DBP:  20, 200, 2,000, and 10,000 ppm.  DINP: 40,400, 4,000, and 20,000 ppm.  DEHA:  480, 2,400, and 12,000  ppm.  Diet | GD 15 to PND 21 | - | PND 140-141 | Sexual behavior | The number of mounts and intromissions is reduced in the males that received the dose of 40 DINP and 480 DEHA. The number of ejaculations was reduced in the DBO group at doses 200 and 2000 and was increased in the dose of 10000. The ejaculations that also reduced in the DINP group at dose 40. In the DEHA group, ejaculations were reduced in the doses of 480 and 12000. The post-ejaculation interval is reduced in the DBP group at dose 10000. | Males | Lee et al. (2006) |
| Wistar  rats | DBP  0, 25, 75, 225, and 675 mg/kg/d  Intragastric gavage | GD 6 to PND 28 | - | PND 30  PND 60  PND 61 | Morris water maze    Hidden platform trial and reverse trial  Probe trial | Male pups treated with high-dose DBP showed shorter latency and path length than control, enhancement in spatial acquisition.  No changes.  Increased in the percentages of the probe time spent in a circle, displayed better retention of spatial memory. | Both | Li et al. (2009) |
| Wistar  rats | DBP  0, 0.037, 0.111, 0.333, and 1%  Powdered diet | GD 8 to PND 28 | - | PND 28 - 30 | Water maze  Open-field | Male  0.037%: Decrease spatial memory acquisition.  0.037 and 0.111%: learning impairments.  No changes. | Both | Li et al. (2009) |
| Wistar  rats | DINP 300,600, 750, and 900 mg/kg/d  Intragastric gavage | GD 7 to PND 17 | - | PND 60  PND120  PND150 | Morris water maze  Sweet preference testing  Radial arm maze | At the end of the learning period and on the first day of memory testing there was a marked sex difference, as swim length and latency to reach the platform were almost twice as long in control females compared to control males.  Males and females intake more saccharin at dose 750.  No changes. | Both | Boberg et al. (2011) |
| Wistar  rats | DEHP  30 mg/kg/d  Drinking water | GD 12 to the day of sacrifice | - | PND 30, 45, and 60 | Elevated Plus Maze | Male: PND 45 and 60 - anxiogenic-like  Female: No changes | Both | Carbone et al. (2013) |
| Sprague Dawley  rats | DEHP  10 mg/kg/d  Intragastric gavage | PND 2 to PND 21 | - | PND 57 and PND 60 | Elevated Plus Maze  Open-field | The percent of open arms entries was reduced in DEHP group compared with control. The time spent in open arms was reduced in DEHP group compared with control. Compared with DEHP group, exercise restored the time spent in open arms in DEX (DEHP + Exercise) group.  No changes | Females | Wang et al. (2014) |
| ICR  Mice | DEHP  10, 50, and 200 mg/kg/ d  Intragastric gavage | GD 7 - PND 21 | - | PND 42 and PND 84 | Open-field                                                Morris water maze | Females (50 mg/Kg/d) had the lowest number of crosses at week 6, while females at (10 and 200 mg/Kg/d) had at week 12. Females (10 and 200 mg/Kg/d) had a decrease in the number of rearings in the 6th week. Females (10 and 50 mg/Kg/d) had an increase in grooming compared to males of the same group and males (200 mg/Kg) had an increase in grooming compared to the control, all in the 6th week. In the 12th week, females (10mg/Kg) increased the number of groomings compared to control and males from the same group.  Females (50 and 200 mg/Kg) remained less time in the central area than males from the same group  At week 6, males at doses of 50 and 200 had longer latency to locate the platform. | Both | Dai et al. (2015) |
| C57BL/6 mice | DEHP 30 mg/kg/d  Intragastric gavage | Eight-week-old for more 4 weeks and in the GD 0 to PND 21 | - | PND 56 | Open-field  Elevated plus maze  Morris water maze  Social interaction test | No changes.  No changes.  No changes.    Maternal DEHP exposure decreased offsprings’ social preference to a strange. | Both | Lee et al. (2015) |
| Sprague Dawley  rats | BBP  10,0 μg /ml  Diet | GD 14 to PND 23 | - | PND 59 | Fear conditioning | Freezing behavior  Male and female: decrease in the tone phase.  Female: decreased in the intertone interval. | Both | DeBartolo et al. (2016) |
| Sprague Dawley  rats | DiPeP  1, 10, and 100 mg/kg/d  Intragastric gavage | GD 10 to PND 21 | - | Elevated plus maze (PND 25-27 and PND 70-73)  Play behaviour (PND 37-40)  Partner preference (PND 82 and 88)  Sexual behaviour (PND 125-140) | Elevated plus maze  Play behavior  Partner preference  Sexual behaviour | No changes.  No changes.  1 and 10 - Did not display any preference difference between male and female compartments.  1- Displayed higher preference for the male compartment.  1- Increase in mount latency and intromission latency. | Males | Neubert da Silva et al. (2019) |
| C57BL/6 mice | DBP  50 and 100 mg /kg/d  Mixed in their food | GD 13 - PND 15 | Indifferent maternal misbehavior, poor nesting and recovery methods | PND 4, 7 and 14 | Negative geotaxy  Cliff avoidance  Swimming test  Olfactory orientation | Male - 50 and 100: decreased  Female - 50: decreased  Male - 50: decreased  Male and female - 50 and 100: decreased  Male - 100: decreased  Female - 50: decreased | Both | Lee et al. (2020) |
| Wistar  rats | Phthalate (DEHP, DINP, and DBP)  4.5 mg/kg/d  Cookie | GD 15 to PND 4 | - | Weaning (PND 21-23);  Puberty (PND 42-44);  Adulthood (PND77-79). | Social interaction  Weaning  Puberty  Adulthood | Frequency of sociocohesive  interactions:  Male and female – Decrease  Female – Decrease  Female – Decrease | Both | Morová et al. (2020) |
| Long Evans rats | Phthalate  0, 200, or 1000 μg/kg/d  Cookie | Perinatal  GD 0 to PND 10  Adolescent (PND 27 to 55) | - | Perinatal – PND 85-90  Adolescent – PND 85, 87, 99, and 112 | Perinatal:  Elevated plus maze  Prepulse inhibition  Adolescent:  Elevated plus maze  Attentional set shift  Prepulse inhibition | No changes.  No changes.  Male /1000 - Decrease in the number of closed arm entries  Female/1000 – Increased trips to the end of the open arm  No changes.  No changes. | Both | Sellinger et al. (2020) |
| Sprague Dawley  rats | DEHP  10 mg/kg/day  Intragastric gavage | Pregnancy (GD 14-21) | - | PND 57-66 | Open-field  T-maze | No changes.  Decrease in the working memory | Males | Wang et al. (2020) |
| ICR  mice | DEHP  200 mg/kg/d  Intragastric gavage | Group 1: GD 6 to GD 12  Group 2: GD 13 to GD 17 | - | PND 42 and PND 56 | Open-field  Elevated plus maze  Morris water maze | No changes.  No changes.  Spatial memory ability was impaired for male rather than female offspring in gestation 13–17 days’ group. | Both | Zhao et al. (2020) |
| Sprague Dawley  rats | DBP: 500 mg/kg  DES: 125 µg/kg  Intragastric gavage | DBP: every second day from GD 14 to PND 6  DES: at GD 14 and GD 16 only | - | PND 60 | Sexual behavior | DBP males presented an increased in the latency to mount. DES females presented reduced locomotion and reduced agonistic behavior compared to controls and DBP group. | Both | Hunter et al. (2021) |

Adm= administration; d= day; GD=gestational day; PND=postnatal day; (-) not analyzed; LD=lactation day; DEHP= diethylhexylphthalate; DINP= diisononyl phthalate; DBP= dibutyl phthalate; DiPeP=diisopentyl phthalate; DEHA= di-(2-ethylhexyl) adipate; BBP= benzyl butyl phthalate; rodent= rat and/or mouse; DES= Diethylstilbestrol.

**Table S3:** EDCs effects on rat intergenerational behavior: Vinclozolin (VIN) studies

| **Species** | **Dose of EDCs used and adm. route** | **Exposure period** | **Behavioral outcomes in dams** | **Time of behavioral analyses in the offspring** | **Behavioral tests performed on offspring** | **Behavioral findings found in offspring** | **Male and/or female** | **References** |
| --- | --- | --- | --- | --- | --- | --- | --- | --- |
| Sprague Dawley  rats | VIN  0, 10, 150, and 750 ppm  In diet | GD 7 to PND 77 | No change in maternal nursing behavior. | PND 22-24  PND 35  PND 63-77  PND 69-75 | Open-Field  Play behavior  Running wheel  Consumption of saccharin | No changes.  No changes.  High-dose females were significantly less active.  Males and females increased consumption. | Both | Flynn et al. (2001) |
| Long Evans rats | VIN  0, 1.5, 3, 6, and 12 mg/kg/d  Intragastric gavage | GD 14 to PND 3 | - | PND 22 and PND 34 | Social play behavior | 12 mg/kg VIN dose significantly increased play behavior in the male offspring on PND 34 compared with controls. | Both | Colbert et al. (2005) |
| Long Evans rats | VIN  0, 1.5, 3, 6 or 12 mg/kg /d  Intragastric gavage | GD 14 to PND 3 | - | PND 60 | Runway procedure | Male offspring required more trials than females. | Both | André and Markowski (2006) |
| Wistar rats | VIN  1 mg/kg/d  Orally (micropipette) | GD 0 to PND 21 | - | PND 25 | Sweet preference | Male offspring consumed a higher volume of saccharin solution. | Both | Kouidhi et al. (2014) |
| Sprague Dawley  rats | VIN  1 mg/kg/d  In diet | GD 8 to GD 18 | - | Lactactional period  Adulthood | Ultrassonic vocalizations (USVs)  Sexual behavior | Reduced vocalizations in females and males.  Males reduced lateral kicks and increased intromission frequency and lordosis quotient, and females from the same group exhibited lower latency to the first enter mating chamber, increased lordosis intensity, increased frequency of mounts and increased latency to first intromission. | Both | Krishnan et al. (2018) |
| Sprague Dawley  rats | VIN  1 mg/kg/d  In diet | GD 8 to GD 18 | - | Lactactional period  Adulthood | Ultrasonic vocalizations (USVs)  Sexual behavior | No changes.  Increased sexual behavior in females (F2). |  | Krishnan et al. (2019) |
| Sprague Dawley  rats | VIN  1 mg/kg/d  In diet | GD 8 to GD 18 | - | PND 60 | Mate preference  Odor preference  Odor discrimination | Adult male offspring had impaired mate preference behavior.  Similar pattern of impairment was observed for urine-soaked filter paper from the same rat group that received VIN.  No changes. | Both | Scudder et al. (2020) |
| Sprague Dawley  rats | VIN  1 mg/kg/d  In diet | GD 8 to GD 18 | - | Adulthood | Sociosexual preference test | Impaired sexual behavior. |  | Scudder et al. (2021) |

Adm= administration; d= day; GD=gestational day; PND=postnatal day; (-) not analyzed.

**Table S4:** EDCs effects on mice transgenerational behavior: BPA studies

| **Species** | **Dose of EDCs used and route of adm.** | **Exposure period** | **Time of behavioral analyses in the offspring** | **Behavioral outcomes in male offspring** | **Behavioral outcomes in female offspring** | **Reference** |
| --- | --- | --- | --- | --- | --- | --- |
| C57BL/6J (B6) mice | Chow supplemented with 5 mg/BPA/kg diet *ad libitum* | 7-10 days prior mating until birth of pups, only female mice in the F0 generation | PND 21 of the F1, F2 and F4 generation | - Increase in social interactions (F2 and F4), while F1 exhibited a decrease (Social Interaction Task);  - No significant effects observed in the Elevated-Plus Maze Test. | - Increase in social interactions (F2 and F4), while F1 exhibited a decrease (Social Interaction Task);  - No significant effects observed in the Elevated-Plus Maze Test. | Wolstenholme et al. (2012) |
| C57BL/6J (B6) mice | Chow supplemented with 5 mg/BPA/kg diet *ad libitum* | 7-10 days prior mating until birth of pups, only female mice in the F0 generation | PND 21 of the F1 and F3 generation | - Increase in social investigation (F1) and decrease in response to a novel stimuli (F3) (Social Recognition Task);  - Increase in locomotor activity (F3) (Open-Field Test);  - Normal olfactory discrimination (F3) (Odor Discrimination Task); | - Increase in social investigation (F1) and decrease in response to a novel stimuli (F3) (Social Recognition Task);  - Increase in locomotor activity (F3) (Open-Field Test);  - Normal olfactory discrimination (F3) (Odor Discrimination Task); | Wolstenholme et al. (2013) |
| C57BL/6J (B6) mice  Friend virus B mice (only to brain collection of F3 generation in another exposure model to BPA in order to explore mechanisms and maternal inheritance) | Chow supplemented with 5 mg/BPA/kg diet *ad libitum* | 10 days prior mating until birth of pups, only female mice in the F0 generation | PND 21-23 of the F3 generation (maternal and paternal lineage) | - Maternal lineage: BPA-exposed had an impair in dishabituation trial with a decreased interaction toward a novel mouse;  - Paternal and maternal lineage: more locomotor activity in the habituation of the Sociability Test. | - Maternal lineage: BPA-exposed had an impair in dishabituation trial with a decreased interaction toward a novel mouse;  -Paternal and maternal lineage: more activity in the habituation of the Sociability Test. | Wolstenholme et al. (2019) |

Adm= administration; GD=gestational days; PND=postnatal days; (-) not analyzed; ED=embryonic day.

**Table S5:** EDCs effects on mouse transgenerational behavior: Phthalates (DEHP) studies

| **Species** | **Dose of EDCs used and route of adm.** | **Exposure period** | **Behavioral outcomes in dams** | **Time of behavioral analyses in the offspring** | **Behavioral outcomes**  **in male offspring** | **Behavioral outcomes in female offspring** | **Reference** |
| --- | --- | --- | --- | --- | --- | --- | --- |
| C57BL/6J mice | 150 and 200 mg/kg of DEHP,  via oral by gavage | GD 7-14, only female mice in the F0 generation | **-** | PND 25-32 of the F3 generation (150 mg/kg group)  PND 35-42 of the F3 generation (200 mg/kg group) | - Increase in the dig time and decrease in the self-grooming time by 200 mg/kg group (Social Interaction Tests), an autism-like phenotype;  - No significant effects observed in the Elevated-Plus Maze Test. | - Not tested in the Social Interaction Tests;  - No significant effects observed in the Elevated-Plus Maze Test. | Quinnies et al. (2015) |
| C57BL/6J mice | 5, 40, and 400 μg/kg of DEHP, via oral by consume of a “cocoa puff” coated in 50 μL of stripped corn oil containing the doses of DEHP | GD 0 until LD 10, only female mice in the F0 generation | - F0 dams did not display any DEHP-related differences in maternal care;  - F2 dams did not show any effects of DEHP dose lineage, but dams spent more time licking and grooming their litters on PND 2 than on PND 4;  - F0 dams spent less time in the nest, licking and grooming pups, and nursing, and spent more time digging in the cage, as compared with the F2 dams. | PND 2, PND 4, and PND 6: behavioral analysis in dams  PND 28-32 (Social Interaction Test)  PND 30-35 (Elevated-Plus Maze Test) | - F3 DEHP-exposed (400 μg/kg group) were highly interactive: more side-by-side sitting behavior and fewer independent behaviors overall, as time of total time exploration of the cage (Social Interaction Test);  - F3 DEHP-exposed (400 μg/kg group) showed an increase in the time spent in the closed arm (Elevated-Plus Maze Test): anxiety-like behavior. | - F3 DEHP-exposed (400 μg/kg group) showed an increase in “sitting alone” and a decrease in the total time exploration of the cage (Social Interaction Test);  - No significant effects observed in the Elevated-Plus Maze Test. | Quinnies et al. (2017) |
| CD-1 mice | 20 μg/kg/day, 200 μg/kg/day, 500 mg/kg/day, and  750 mg/kg/day of DEHP, oral route by pipette | GD 10.5 until birth of the pups (GD 19.5), only female mice in the F0 generation | **-** | PND 90-100 of the F3 generation | **-** No significant effects observed in the Elevated-Plus Maze Test. | **-** Increase in total time spent in the open arms by 750 mg/kg/day DEHP-exposed  female group (Elevated-Plus Maze Test): anxiolytic-like behavior. | Hatcher et al. (2019) |

Adm= administration; GD=gestational days; PND=postnatal days; (-) not analyzed; ED=embryonic day; LD=lactation day; DEHP= di-(2-ethylhexyl) phthalate.

**Table S6:** EDCs effects on rat transgenerational behavior: Vinclozolin (VIN) studies

| **Species** | **Dose of EDCs used and route of administration** | **Exposure period** | **Behavioral outcomes in dams** | **Time of behavioral analyses in the offspring** | **Behavioral outcomes**  **in male offspring** | **Behavioral outcomes in female offspring** | **Reference** |
| --- | --- | --- | --- | --- | --- | --- | --- |
| Sprague-Dawley rat | 100 mg/kg dose of VIN, daily, IP injections | GD 8-14, only female rat in the F0 generation | - | PND 90-120 of the F3 generation | **-** Male F3 no exhibited preferences for female exposed or not-vinclozolin in the F0 (Mate-Preference Behavioral Analysis);  - Male F3 vinclozolin-exposed spent more time exploring not-vinclozolin exposed female in F0 (Odor-Salience Analysis). | - Female F3 exhibited greater preference for male not-vinclozolin exposed in the F0 (Mate-Preference Behavioral Analysis);  - Female control F3 did not spend more time exploring vinclozolin-exposed male in the F0 (Odor-Salience Analysis) | Crews et al. (2007) |
| Sprague-Dawley rat | 100 mg/kg dose of VIN, daily, IP injections | GD 8-14, only female rat in the F0 generation | - | Females designated as ‘‘young’ (PND 93-124), males designated as ‘‘young’’ (PND 82-155), females designated as ‘‘aged’’(PND 369-386), and males designated as ‘‘aged’’ (PND 202-385) | - “Young” exposed-vinclozolin male rats showed an increase in the time spent on the light side of the box and in the number of transitions between the light and dark sides of the box (Light:Dark Box) and an increase in the number of total arm entries (Elevated Plus-Maze Test). | - “Aged” exposed-vinclozolin female rats showed a tendency for lower latency to enter the dark side and a significant  decrease in time spent in the light side of the box, with no  differences in the number of transitions (Light:Dark Box)  - “Aged” and “young” exposed-vinclozolin female rats showed a decrease in both the percent time spent on the open arms and in the percent of open arm entries, with no difference in the number of total arm entries (Elevated Plus-Maze Test). | Skinner et al. (2008) |
| Sprague-Dawley rat | 100 mg/kg dose of VIN, daily, IP injections | GD 8-14, only female rat in the F0 generation | - | Chronic restraint stress (CRS) during adolescence in the male rats (PND 26-46)  PND 114-118 of the male F3 generation | - VIN-exposed male submitted to CRS in adolescence showed increased anxiety behavior in adulthood (Open-Field Test);  - No-stress VIN-exposed males visited the stimulus animal for longer periods and move more between chambers in the Sociability Test (Soc 1);  - VIN-exposed males showed effects of stress, traveling farther and faster than VIN no-stress males; and also spent less time in the center compartment and more time with the familiar and novel stimulus males in the Social Test (Soc 2). | - | Crews et al. (2012) |
| Sprague-Dawley rat | 100 mg/kg dose of VIN, daily, IP injections | GD 8-14, only female rat in the F0 generation | - | Chronic restraint stress (CRS) during adolescence (PND 23-44)  PND 90-118 of the F3 generation | - Male exposed to CRS in adolescence showed decreased anxiety behaviors in adulthood (Open-Field Test and Light:Dark Box);  - Higher preference to associate with an animal as opposed to an empty cage and no preference for social novelty (Sociability and Social Novelty Test). | -No differences caused by CRS were found in females (Open-Field Test and Light:Dark Box);  - Higher preference to associate with an animal as opposed to an empty cage and spent more time with a novel animal opposed to a familiar one (Sociability and Social Novelty Test). | Gillette et al. (2014) |
| Sprague-Dawley rat | 100 mg/kg dose of VIN, daily, IP injections | GD 8-14, only female rat in the F0 generation | - | 11 months of age (F3 generation) | - Male F3 generation VIN-lineage: spent less time in the closed arm and there was a trend toward them spending more time in the open arm of the Elevated-Plus Maze Test, exhibiting an increased in the locomotor activity, not confirmed in the Open-Field Test. | - Not showed locomotor or anxiety-related alterations in the Elevated-Plus Maze and Open-Field Tests. | Nilsson et al. (2019) |
| Sprague-Dawley rat | PCB mixture Aroclor 1221 (PCB group) or VIN (VIN group), both at 1 mg/kg, IP  injections | GD 8-18, only female rat in the F0 generation and formation of maternal and paternal lineage | - Maternal lineage F2 dams (PND 3 at lights-on phase): decrease in active nursing position (PCB group), and increase in the numbers of observations of licking and grooming (VIN group).  - Paternal lineage F2 dams (PND 3 lights-on phase): decrease in the number of observations on the nest and in the observations passive nursing (PCB group);  - Maternal lineage F2 dams (PND 3 at dark phase ): increase in numbers of observations on the nest (VIN group);  - Paternal lineage F2 dams (PND 3 at dark phase): increase in numbers of observations nursing and active nursing position (VIN group). | PND 3 and PND 6: F3 pup ultrasonic vocalizations (maternal and paternal lineage)  PND 75-100 of the F3 generation (maternal and paternal lineage) | - Maternal lineage pups: decrease in call durations on PND 6 (VIN group);  -Paternal lineage pups: decrease in total and frequency-modulated calls on PND 6 (PCB group);  - Maternal lineage F3: VIN males moved more quickly, but spent less time immobile, suggesting lower levels  of stress reactivity (Open-Field Test); | - Maternal lineage pups: decrease in call durations on PND 6 (VIN group);  -Paternal lineage pups: decrease in total and frequency-modulated calls on PND 6 (PCB group);  - Maternal lineage F3: VIN females moved more quickly and spent less time in the center of the chamber, suggesting higher stress reactivity (Open-Field Test);  - Paternal lineage F3: VIN females showed an increased time immobile (Light:Dark Box). | Krishnan et al. (2019) |

GD=gestational days; PND=postnatal days; (-) not analyzed; ED=embryonic day; IP (intraperitoneal); VIN (vinclozolin); PCBs (polychlorinated biphenyl); CRS (chronic restraint stress).
